# Large Deflection Analysis of Bimodular Functionally Graded Truncated Thin Conical Shells Under Mechanical and Thermal Loads

**DOI:** 10.3390/ma18020362

**Published:** 2025-01-14

**Authors:** Xiao-Ting He, Ming-Wei Luo, He-Hao Feng, Jun-Yi Sun

**Affiliations:** 1 School of Civil Engineering, Chongqing University, Chongqing 400045, China; 202216021079@stu.cqu.edu.cn (M.-W.L.); 202216131289t@stu.cqu.edu.cn (H.-H.F.); sunjunyi@cqu.edu.cn (J.-Y.S.); 2 Key Laboratory of New Technology for Construction of Cities in Mountain Area (Chongqing University), Ministry of Education, Chongqing 400045, China

**Keywords:** large deflection, truncated thin conical shell, bimodular effect, functionally graded materials, thermal load

## Abstract

The purpose of this study is to analyze the large deflection problem of bimodular functionally graded truncated thin conical shells under the transverse mechanical load and non-uniform thermal load, in which two different boundary constraints of the truncated shell with two ends simply supported and fully fixed are considered. It is assumed that the temperature distribution along the thickness direction satisfies the Fourier law of heat transfer, and the material properties change exponentially along the thickness direction while different properties in tension and compression are considered. The geometric equation of the conical shell is established based on the equivalent method of curvature correction of von-Kármán deformation theory, and the analytical solution of the problem is obtained by Ritz method. Numerical simulation of bimodular functionally graded conical shells under the thermal and mechanical loads is carried out by Abaqus, and the numerical solution agrees with the theoretical solution. The results show that the introduction of bimodular functionally graded material will affect the maximum displacement and this effect has different rules under the mechanical load and thermal load. In addition, factors such as the cone apex angle and the truncated distance have a great influence on the maximum displacement and its location of the conical shell.

## 1. Introduction

Conical shells, as a common structure type, are widely used in civil engineering, mechanical engineering, aerospace engineering, and marine engineering, for example, silos [1], ship bows [2], and missile and spacecraft components. An increasing number of advanced composite materials are being utilized in the construction of these structures. Among them, functionally graded materials (FGMs) are extensively employed in high-temperature service structures due to their exceptional properties, including high strength, good toughness, high temperature resistance, and resistance to thermal stress concentration [3,4,5]. A shell structure has the characteristics of making full use of the strength of pressure-resistant materials, it is easy to obtain in a large space, it has a large bearing capacity brought by curvature characteristics, has a beautiful shape, and so on, which are favored by engineers [6]. However, thin shells under heavy loads are often prone to large deflection and stability problems, so the study of thin shells made of various new composite materials has become a research hotspot in recent years [7].

FGMs were originally developed as heat-shielding material for aerospace structural applications and fusion reactors. FGMs are a mixture of two or more materials in which the volume fractions (VFs) are continuously varied depending on the position along a certain dimension to achieve the desired function. For instance, thermal barriers for high temperature applications can be formed from a mixture of ceramics and metal [8,9,10]. However, previous studies have shown that the elastic modulus of ceramics and metals is not the same under tension and compression, and the researchers overlooked the different mechanical properties which FGMs may exhibit under tension and compression, namely, the bimodular effect [11]. With the development of materials science, the preparation cost of FGMs has gradually decreased, so this well-performing composite has been applied in numerous engineering fields [12]. In fact, whether significant or not, the bimodular effect should be prevalent in almost all materials, but due to the complexity of analysis caused by the introduction of bimodular effects, this property has been overlooked in most studies [13,14]. The purpose of this study is to analyze the large deflection problem of bimodular FGM conical shells under mechanical and thermal loads. Therefore, the next step is to elaborate on the bimodular material, the large deflection of plates and shells, and finally, the structural analysis of conical shells.

Bimodular material refers to the material with different elastic moduli in tension and compression [15]. In order to describe the mechanical behavior of this material, many reliable models have been proposed, and there are two widely used models in the engineering field. One is the anisotropic material model proposed by Bert [16], which is applied to the study of composite materials reinforced by fibers, whose tensile and compressive elastic moduli are judged on the longitudinal strain of fibers [17,18]. The other is the isotropic material model proposed by Ambartsumyan [12], whose tensile and compressive elastic moduli are judged on the positive and negative signs of the principal stresses. Based on Ambartsumyan’s material model, extensive works on bimodular functionally graded structures have been carried out, including bimodular FGM beams [19], thin plates [20,21], cylindrical shells [22], curved beams [23], and the thermal stress problem of bimodular FGM curved beams [24].

In recent decades, many achievements have been made in the study on the large deflection of functionally graded plates and shells, whether analytical or numerical. Based on von Kármán’s large deformation theory, Woo and Meguid [25] studied the large deflection buckling of functionally graded plates and shells under a lateral load and temperature field, and obtained Fourier series solutions. Na and Kim [26] used the 3D finite element method to study the nonlinear bending of functionally gradient plates under the combined action of uniform load and thermal load. Van Tung [27] used the Galerkin method to solve the nonlinear buckling problem of FGM shallow spherical shells. Wan and Li [28] studied the thermal buckling behavior of FGM cylindrical shells by converting the governing equations based on Donnell shell theory into ordinary differential equations using the method of separating variables. Sahmani and Aghdam [29] examined the axial buckling and post-buckling response of cylindrical nanoshells made of FGMs in the presence of surface free energy effects. Golmakani and Alamatian [30] studied the nonlinear flexural behavior of radial functionally graded (RFG) annular plates of medium thickness; the nonlinear formulations were developed based on first order shear deformation theory (FSDT) using the von-Kármán theory for large deflections and including the plate-foundation interaction. Based on the third-order shear deformation theory and von-Kármán theory, Vuong and Duc [31] proposed an analysis method for static and dynamic stability and nonlinear vibration of functionally graded torus shell sections. Tornabene and Ceruti [32] investigated the large deflection problem of thin plates subjected to lateral pressure loads and relatively thick rectangular functionally graded plates, and obtained the solution by minimizing the total potential energy. In addition, the results of finite element studies on functionally graded shells are richer than the analytical results. With the innovation of computer technology, more complex problems have been solved. As early as 20 years ago, some finite element formulas for predicting the variation characteristics of FGMs were developed, such as the penetration thickness integral method [33], the high-order Gaussian integral method [34], estimating bidirectional functional gradient material properties by traditional average method [35], and using special mixing or Voronoi element finite element analysis [36,37]. Naghdabad and Kordkheili [38] derived the finite element formula for thermoelastic analysis of FGM plates and shells. In the past 20 years, the finite element analysis of the large deflection of functionally graded shells has undergone technical optimization [39,40,41], algorithm extension [42,43] and further improvement of shell elements [44,45,46]. Hernández et al. [47] conducted a comprehensive review of the development of the finite element method of functionally graded shells.

For the study of conical shell problems, the earliest literature can be found in the paper by Lukasiewicz and Szyszkowski [48], who analyzed the post-buckling behavior of isotropic cylindrical shell and conical shell under axial compression according to the Lagrange variational principle. Wang et al. [49] used the two-parameter perturbation method to solve the large deflection problem of a shallow conical shell under asymmetric loads, for the first time. Many achievements have been made in the study of isotropic conical shells. For example, based on the classical thin shell theory, Liew et al. [50] studied the free vibration of thin conical shells with different boundary conditions by using the element-free kp-Ritz method. Sofiyev and Aksogan [51] solved the dynamic buckling problem of conical thin shell with variable thickness by Ritz type variational method. Zielnica [52] studied the static stability of elastoplastic conical shells. Abundant research results have also been obtained on FGM conical shells. Bhangale et al. [53] conducted linear thermal buckling analysis on conical shells with functional gradient truncated tops at different half-vertex angles based on the semi-analytic finite element method. Based on classical linear shell theory, Zhang et al. [5] obtained an accurate solution of thermoelastic bending of functionally graded conical shells. Duc and Cong [54] studied the thermal stability of an eccentrically reinforced functionally gradient truncated conical shell surrounded by an elastic foundation. Sofiyev [55] studied the vibration and elastic and thermoelastic stability of FGM conical shells under different static and dynamic loads, and systematically reviewed the research on FGM conical shells in the past two decades [3]. In the past decade, with the maturity of computer technology, finite element methods for thin shell problems have been greatly developed, such as isogeometric analysis (IGA). Bazilevs et al. [56] introduced a new formulation for modeling progressive damage in laminated composite structures based on the isogeometric analysis and Kirchhoff–Love thin shell. Nguyen-Thanh et al. [57] presented a new concurrent simulation approach to couple isogeometric analysis with the meshfree method for the studying of crack problems. The proposed approach is also applied to simulate the crack propagation under a mixed-mode condition. Vu-Bac et al. [58] proposed a novel formulation that tackles, using both nonlinear kinematics and material models, with the coupling between elasticity and solvent transport using Kirchhoff–Love shell theory discretized using isogeometric analysis. Milić et al. [59,60] dealt with the isogeometric analysis of active composite laminates with piezoelectric layers, and used NURBS basis function to establish an equigeometric shell formula based on Reissner–Mindlin kinematics. In addition, deep neural networks (DNN) have a powerful role in predicting the uncertainty of shell deformation, for example, Pan et al. [61] proposed a data-driven solution based on a deep neural network with input layer, hidden layer and output layer, independent variables, and other relevant parameters when studying the thermal stress response of cylindrical shells under a thermal shock load on elastic substrates. It provided a low computational cost method for solving such engineering problems.

According to the above review concerning bimodular material, large deflection problems of plates and shells, and the structural analysis of conical shells, it is found that most scholars often ignored the influence of a bimodular effect on functionally graded thin conical shells, and such a neglect may lead to large errors for structures requiring a refined analysis. At the same time, the introduction of a bimodular effect on materials and the consideration of structural large deformation will inevitably increase the complexity in the analysis. The problem will be challenging for the possible difficulties arising from a physical equation and geometrically nonlinear relation.

In this paper, the large deflection problem of bimodular FGM-truncated thin conical shells under different boundary constraints and subjected to thermal and mechanical loads is studied theoretically and numerically. The structure of this paper is as follows: In Section 2, the constitutive relation of FGMs with different moduli in tension and compression is described, and the formula of the neutral layer of bimodular FGM structures under thermal load is determined. Section 3 is the derivation of the geometric equation for the large deflection of conical shell. In Section 4, the thermoelastic strain energy of conical shell is obtained, and the analytical solutions for FGM conical shells with two ends simply supported and fixed are obtained by Ritz method. In Section 5, computational examples are given and numerical simulation is carried out based on Abaqus. In Section 6, the influences of many factors from the material, geometry, and load aspects on deformation of the conical shell are discussed in detail. Finally, Section 7 is the conclusion.

## 2. Bimodular Functionally Graded Material Model

### 2.1. Constitutive Model

**Figure 1:**
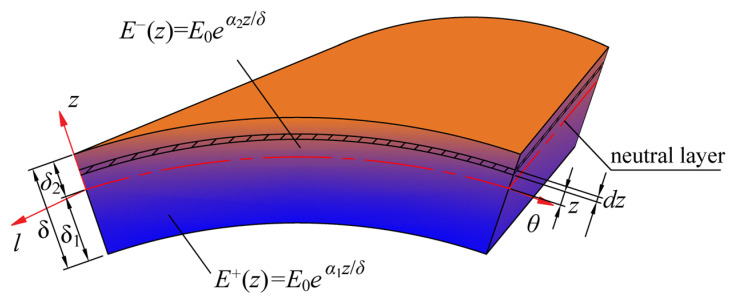
Schematic diagram of material properties and neutral layer along thickness direction of bimodular FGM conical shell.

According to a simplified mechanical model based on our earlier works for bimodular functionally graded materials [14], the elastic modulus of structural materials is assumed to be in the form of an exponential function that distinguishes tension and compression:

(1) $$E^{+}(z)=E_{0}e^{\alpha_{1}z/\delta },E^{-}(z)=E_{0}e^{\alpha_{2}z/\delta },$$

where *E*_0_ is the elastic modulus of the neutral layer materials of the shell, $\alpha_{1}$ and *α*_2_ represent two different gradient indices, *z* is the distance from one point in the shell to the neutral layer, and *δ* represents the thickness of the shell, as shown in Figure 1, in which, *l* and *θ* represents the generatrix direction and circumferential direction, respectively. Within the tensile zone (−*δ*_1_ < *z* < 0), the elastic modulus is *E*^+^(*z*), and in the compressive zone (0 < *z* < *δ*_2_), the elastic modulus is *E*^−^(*z*). Due to the introduction of bimodular property, the neutral layer of the structure (the surface composed of the points where the normal stress is zero) is no longer in the middle of the thickness, namely, *δ*_1_ ≠ *δ*_2_, and their specific calculation formula will be elaborated on later. In fact, even in the absence of the bimodular effect, the neutral layer is generally not located in the middle of the section only due to the FGMs. In addition, it is necessary to distinguish the Poisson’s ratios in tension and compression; thus, *μ*^+^ and *μ*^−^ are considered here.

### 2.2. Non-Uniform Heat Distribution Along Thickness Direction

The formulation of thermal stress has been mentioned in many papers, for example, article [5], and in this study, it is assumed that the temperature is only conducted along the thickness direction of the shell. The thermal stress *σ_t_* is in the form of

(2) $$\sigma_{t}=\frac{E^{-}(z)}{1-\mu^{-}}\alpha (z)\left[T(z)-T_{0}\right],$$

where *α*(*z*) is the coefficient of thermal expansion, *T*(*z*) is the heating function, both changing only with *z*, and *T*_0_ as the initial ambient temperature which will be given in the next computation, for example, *T*_0_ = 20 °C. It is worth noting that under the constraints of two ends, the temperature rise will inevitably cause compressive stress in the conical shell, so the corresponding elastic modulus and Poisson’s ratio are *E*^−^(*z*) and *μ*^−^. It is assumed that the coefficient of thermal expansion *α*(*z*) has the following function form:

(3) $$\alpha (z)=\alpha_{0}e^{\alpha_{3}z/\delta },$$

where $\alpha_{0}$ is the coefficient of thermal expansion at the neutral layer, and *α*_3_ is another gradient index. Note that the function selection for *α*(*z*) is based on the following two considerations: first, it should preferably be consistent with the functionally graded function, like Equation (1), and second, it is also convenient to make mathematical operations, especially for integration and differentiation. Therefore, in Equation (3), we adopt the exponential function. Not only that, for the next coefficient, we will again select exponential functions based on the same considerations.

The heat distribution *T*(*z*) must satisfy Fourier’s law of heat transfer. Assuming that the heat conduction process is stable and there is no internal heat source, a one-dimensional differential equation for heat conduction will give

(4) $$\frac{d}{dz}\left(K(z)\frac{dT(z)}{dz}\right)=0,$$

where *K*(*z*) denotes the thermal conductivity coefficient, and it is assumed that its form coincides with the coefficient of thermal expansion, that is,

(5) $$K(z)=K_{0}e^{\alpha_{3}z/\delta },$$

where *K*_0_ is defined as the thermal conductivity coefficient of the neutral layer. Then, the expression for the heat distribution is obtained by integrating Equation (4):

(6) $$T(z)=-\frac{A_{T1}\delta }{\alpha_{3}K_{0}}e^{-\alpha_{3}z/\delta }+A_{T2},$$

where *A*_T1_ and *A*_T2_ are two integration constants. If here we assume two boundary conditions, namely, *T*(*δ*_2_) = *T*_1_ and *T*(−*δ*_1_) = *T*_2_, the two constants can be solved as follows:

(7) $$A_{T1}=\frac{\alpha_{3}K_{0}(T_{1}-T_{2})}{\delta (e^{-\alpha_{3}\delta_{2}/\delta }-e^{-\alpha_{3}\delta_{1}/\delta })},A_{T2}=T_{1}-\frac{e^{-\alpha_{3}\delta_{2}/\delta }(T_{1}-T_{2})}{e^{-\alpha_{3}\delta_{2}/\delta }-e^{-\alpha_{3}\delta_{1}/\delta }}.$$

### 2.3. Position of the Neutral Layer

In our previous study [14], there has been formulas for determining the neutral surface, but in this study, the non-uniform distribution of heat along the thickness direction is considered, which changes the position of the neutral surface, so it has to be redetermined.

To maintain the balance of forces acting on the cross-section along the thickness direction of the conical shell, it is necessary to ensure that the sum of stresses on the cross-section is zero. However, the membrane stress only causes tension and compression of the cross-section, and does not cause deflection of the cross-section. Therefore, it is only necessary to make the integral of the bending stress acting on the cross-section zero. Based on this idea, the stress balance equations for the two directions, the generatrix direction *l* and circumferential direction *θ*, are established as follows:

(8) $$\left\{\begin{array}{l} \int_{-\delta_{1}}^{0}(\sigma_{l}^{+}-\sigma_{t})dz+\int_{0}^{\delta_{2}}(\sigma_{l}^{-}-\sigma_{t})dz=0\\ \int_{-\delta_{1}}^{0}(\sigma_{\theta }^{+}-\sigma_{t})dz+\int_{0}^{\delta_{2}}(\sigma_{\theta }^{-}-\sigma_{t})dz=0 \end{array}\right.,$$

where $\sigma_{l}^{+}$, $\sigma_{l}^{-}$, $\sigma_{\theta }^{+}$, and $\sigma_{\theta }^{-}$ represent the tensile and compressive stresses along the generatrix direction *l* and circumferential direction *θ*. According to the definition in two-dimensional shell theory [62], their expressions are

(9) $$\left\{\begin{array}{l} \sigma_{l}^{+}=\frac{E^{+}(z)z}{1-{\left(\mu^{+}\right)}^{2}}(\chi_{l}+\mu^{+}\chi_{\theta }),\sigma_{l}^{-}=\frac{E^{-}(z)z}{1-{\left(\mu^{-}\right)}^{2}}(\chi_{l}+\mu^{-}\chi_{\theta })\\ \sigma_{\theta }^{+}=\frac{E^{+}(z)z}{1-{\left(\mu^{+}\right)}^{2}}(\chi_{\theta }+\mu^{+}\chi_{l}),\sigma_{\theta }^{-}=\frac{E^{-}(z)z}{1-{\left(\mu^{-}\right)}^{2}}(\chi_{\theta }+\mu^{-}\chi_{l}) \end{array}\right.,$$

where $\chi_{l}$, *χ_θ_* are defined as the curvature variation of the shell in two directions. They are represented as

(10) $$\chi_{l}=-\frac{d^{2}w}{dl^{2}},\chi_{\theta }=-\frac{1}{l}\frac{dw}{dl}.$$

Although the material is special, it still meets the requirement of isotropy in both directions of the shell. Therefore, the thermal stress at a certain point on the shell is always the same in all directions; Equation (8) can be changed as

(11) $$\left\{\begin{array}{l} \int_{-\delta_{1}}^{0}\sigma_{l}^{+}dz+\int_{0}^{\delta_{2}}\sigma_{l}^{-}dz-\int_{-\delta_{1}}^{\delta_{2}}\sigma_{t}dz=0\\ \int_{-\delta_{1}}^{0}\sigma_{\theta }^{+}dz+\int_{0}^{\delta_{2}}\sigma_{\theta }^{-}dz-\int_{-\delta_{1}}^{\delta_{2}}\sigma_{t}dz=0 \end{array}\right..$$

By subtracting the two integrations in Equation (11) and also substituting Equation (10) into the obtained equation, we have

(12) $$\left(\int_{-\delta_{1}}^{0}\frac{E_{0}e^{\alpha_{1}z/\delta }}{1+\mu^{+}}zdz+\int_{0}^{\delta_{2}}\frac{E_{0}e^{\alpha_{2}z/\delta }}{1+\mu^{-}}zdz\right)\left(\chi_{l}-\chi_{\theta }\right)=0.$$

To satisfy the above equation, first we may have

(13) $$\chi_{l}-\chi_{\theta }=-\frac{d^{2}w}{dl^{2}}+\frac{1}{l}\frac{dw}{dl}=0.$$

In general mechanics of materials, it is known that the first-order derivative of deflection, d*w*/d*l*, represents the rotation angle when it is related only to rotation due to bending, and the second-order derivative of deflection, d^2^*w*/d*l*^2^, is proportional to the bending moment. However, in the shell, there is almost no such point whose rotation angle and the bending moment are zero simultaneously, which indicates that the assumption in Equation (13) is not valid, and thus, we can only obtain

(14) $$\int_{-\delta_{1}}^{0}\frac{e^{\alpha_{1}z/\delta }}{1+\mu^{+}}zdz+\int_{0}^{\delta_{2}}\frac{e^{\alpha_{2}z/\delta }}{1+\mu^{-}}zdz=0.$$

Moreover,

(15) $$\delta =\delta_{1}+\delta_{2},$$

by combining the above two equations with only two unknowns *δ*_1_, *δ*_2_, the neutral surface position of the shell under thermal load can be determined.

## 3. Geometric Equations of Axisymmetric Conical Shells

### 3.1. Coordinate Definition

**Figure 2:**
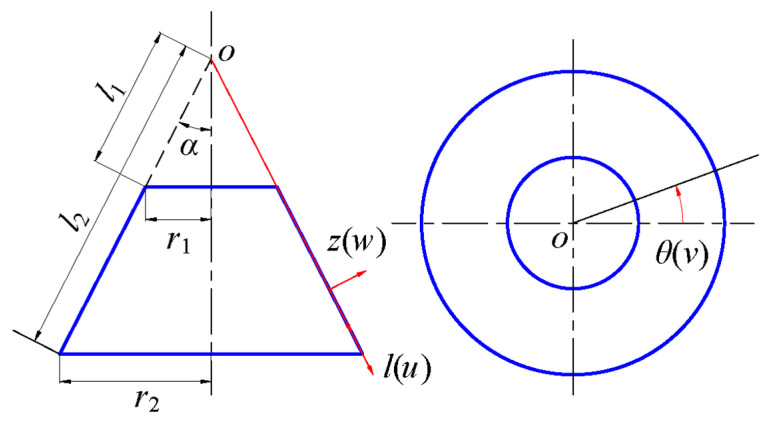
Coordinate system and geometric relation of truncated conical shell.

The object of this study is a truncated thin conical shell that is subjected to both axisymmetric thermal loads and uniformly distributed transverse loads. An orthogonal curvilinear coordinates system (*l*, *θ*, *z*) is established on the neutral layer of the truncated conical shell, as shown in Figure 2, in which, the axis along the generatrix direction is the *l* axis, the axis along the thickness direction is the *z* axis, and the direction of counterclockwise rotation around the symmetry axis of the truncated conical shell is the *θ* axis, and the corresponding displacements along the three directions are *u*, *v*, and *w*, respectively. Other geometric parameters are defined as follows: the half of cone apex angle is *α*, the distance between the cone top and the small end of the conical shell (truncated distance) is *l*_1_, the distance from the small end to the large end (shell length) is *l*_2_, the thickness of the shell is constant, which is denoted by *δ* (see Figure 1), $r_{1}$ and *r*_2_ is the radius of the small end and large end of the conical shell, respectively.

### 3.2. Geometric Equation

In the theory of the small deflection of shells, the influence of the middle surface deformation on the final results is ignored, which is appropriate when the normal deformation of the middle surface, namely the deflection, is much smaller than its thickness. However, when the deflection of the shell reaches the same order of thickness, the influence of the middle surface deformation on the governing equation must be taken into consideration [62].

The nonlinear term of the large deflection equation for thin shells mainly comes from a geometric equation. Therefore, the geometric equation in large deflection bending theory will be obtained by modifying the geometric equation in small deflection bending theory. The small deflection equation of the shell is obtained without considering the influence of the deformation on the geometric equation. However, in the case of large deflection, the elongation of the middle surface caused by normal displacement should be considered. Due to the fact that thin conical shells are a special form of rotating shells, they still satisfy the basic assumptions for rotating shells and thin shells. First, the normal strain along *z* direction is assumed to be zero; second, for truncated conical shells, the geometry, and mechanical and thermal loading, as well as kinematic boundary conditions are all axisymmetric, only then the deformation will also be axisymmetric, indicating that all shear strains are zero.

**Figure 3:**
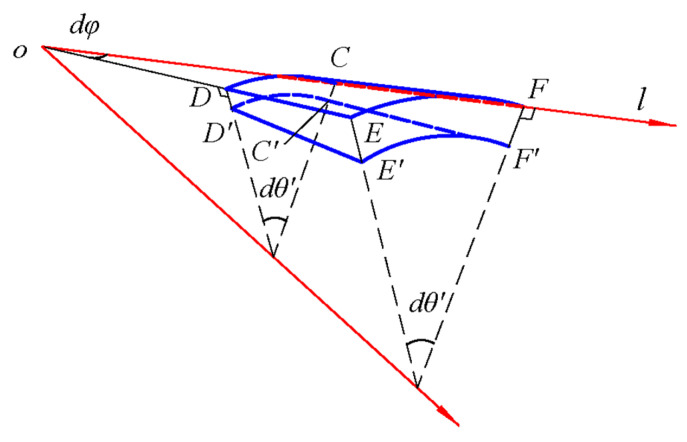
Deformation of the middle surface of the conical shell.

As shown in Figure 3, *CDEF* represents the initial position of the differential element on the middle surface, in which the length of *CF* and *CD* is d*l* and *l∙*d*φ*, respectively. *C′D′E′F′* represents the changed position of the differential element on the middle surface caused by normal displacement *w*. Among them, *CC′* and *DD′* have the same length, indicating that the normal displacement of the conical shell under axisymmetric load is independent of the angle. *CC′* and *FF′* have different lengths, indicating that the normal displacement changes along the generatrix direction *l*. Note that the rotation angle of *C′F′* with respect to *CF* is d*w*/d*l*, thus the length difference of *CC′* and *FF′* will give d*l*·(d*w*/d*l*). Therefore, the strain along the *l* direction caused by the normal displacement *w* may be computed as, by definition,

(16) $$\varepsilon_{l}^{\prime }=\frac{\sqrt{{\left(FF\prime -CC\prime \right)}^{2}+CF^{2}}-CF}{CF}=\frac{dl\sqrt{1+{\left(dw/dl\right)}^{2}}-dl}{dl}\approx \frac{1}{2}{\left(\frac{dw}{dl}\right)}^{2}.$$

Due to the axisymmetric nature of the load and structure, the influence of normal displacement on the circumferential strain *ε_θ_* has been considered in the small deflection theory. The small deflection equation for conical shells has been applied in previous study [5]. Combining with Equation (10), the in-plane and out-of-plane geometric equations for the large deflection of conical shells give

(17) $$\left\{\begin{array}{l} \varepsilon_{l}=\frac{du}{dl}+\frac{1}{2}{\left(\frac{dw}{dl}\right)}^{2},\varepsilon_{\theta }=\frac{u}{l}+\frac{w}{l}\cot\alpha \\ \chi_{l}=-\frac{d^{2}w}{dl^{2}},\chi_{\theta }=-\frac{1}{l}\frac{dw}{dl} \end{array}\right.,$$

which may be used for the next variation method.

## 4. Application of Variation Method

### 4.1. Basic Formulas for Strain Energy and Thermal Stress Work

According to the first law of thermodynamics, the energy stored in an elastic body is called strain energy when the work conducted by an external force is completely transformed into deformation. It is assumed that the elastic body is only subjected to a uniform normal stress *σ*_*l*_ along a certain direction *l*, and the corresponding normal strain is $\varepsilon_{l}$, the strain energy density, that is, the strain energy per unit volume, can be expressed as

(18) $$v_{\varepsilon }=\int_{0}^{\varepsilon_{l}}\sigma_{l}d\varepsilon_{l}.$$

When the stress–strain relationship is linear, that is,

(19) $$\sigma_{l}=E\varepsilon_{l},$$

substituting it into the expression of strain energy density yields

(20) $$v_{\varepsilon }=\int_{0}^{\varepsilon_{l}}E\varepsilon_{l}d\varepsilon_{l}=\frac{1}{2}\sigma_{l}\varepsilon_{l}.$$

According to the law of conservation of energy, the amount of strain energy is not related to the order of forces acting on the elastic body, but is completely determined by the final magnitude of stress and strain. Therefore, it is assumed that in a spatial problem, for example, in a shell whose curvilinear coordinates system (*l*, *θ*, *z*) is established, six stress components and six strain components increase proportionally to the final size, the strain energy density of each stress component can be simply calculated, and then added together to obtain the total strain energy density

(21) $$v_{\varepsilon }=\frac{1}{2}\left(\sigma_{l}\varepsilon_{l}+\sigma_{\theta }\varepsilon_{\theta }+\sigma_{z}\varepsilon_{z}+\tau_{l\theta }\gamma_{l\theta }+\tau_{lz}\gamma_{lz}+\tau_{\theta z}\gamma_{\theta z}\right).$$

In order to obtain the strain energy of the entire elastic body, *U_ε_*, we only need to integrate the strain energy density within the volume *V*, that is,

(22) $$U_{\varepsilon }=\int_{V}v_{\varepsilon }dV,$$

where d*V* = d*l* ∙ *l* ∙ d*φ* ∙ d*z*, according to Figure 2. However, the above analysis only applies to elastic deformation under external mechanical loads. The analysis of strain energy under thermal loads is different. Again, let us start with a one-dimensional problem. It is assumed that the thermal effect only causes deformation along one direction, and the thermal stress–strain relation is

(23) $$\sigma_{t}=E\alpha T,$$

where *α* is the coefficient of thermal expansion and *T* is the temperature rise, and the corresponding thermal strain *ε*_*t*_ = *αT*, thus we have the thermal strain energy density *v*_*t*_

(24) $$v_{t}=\int_{0}^{\varepsilon_{t}}E\alpha Td\varepsilon_{t}=E\alpha T\varepsilon_{t}=\sigma_{t}\varepsilon_{t}.$$

Note that compared to Equation (20), there is no factor of 1/2 in Equation (24). Since in a thermal problem, only linear extension and shortening are considered, and without any shear strains, the total thermal strain energy density in a spatial problem (curvilinear coordinates system (*l*, *θ*, *z*)) will be

(25) $$v_{t}=\sigma_{t}\varepsilon_{l}+\sigma_{t}\varepsilon_{\theta }+\sigma_{t}\varepsilon_{z}.$$

In isotropic materials, the elastic modulus and thermal expansion coefficient along all directions at a certain point are the same, so the thermal stresses along the *l*, *θ*, and *z* directions are the same. Therefore, the work carried out by thermal stress, *W_T_*, is

(26) $$W_{T}=\int_{V}v_{t}dV.$$

### 4.2. Elastic Strain Energy of Conical Shell U_ε_

According to the basic theory of shells, the total strain energy of the conical shell can be equivalent to the superposition of the strain energy *U_ε_*_1_ of the neutral surface deformation and the strain energy *U_ε_*_2_ of the bending deformation, that is,

(27) $$U_{\varepsilon }=U_{\varepsilon 1}+U_{\varepsilon 2}.$$

Due to the assumptions of thin shells, the effects of normal strain and shear force perpendicular to the neutral surface are ignored. Therefore, the strain energy of neutral surface deformation can be expressed as

(28) $$U_{\varepsilon 1}=\frac{1}{2}\underset{V}{∭}(\sigma_{l}\varepsilon_{l}+\sigma_{\theta }\varepsilon_{\theta }+\tau_{l\theta }\gamma_{l\theta })dV.$$

Further considering the symmetry of the structure and load action, we have

(29) $$\tau_{l\theta }=0,\gamma_{l\theta }=0.$$

In addition, the generatrix along the *l* direction of the conical shell is a straight line, so when an external load is applied, the stress along the generatrix direction of the conical shell is tensile, indicating that the elastic constants may take positive quantities, *E*^+^(*z*) and *μ*^+^, while the circumferential direction *θ* is convex outward, thus the stress along this direction is compressive, indicating that the corresponding elastic constants may take negative quantities, *E*^−^(*z*) and *μ*^−^. Therefore, the membrane stress along the two directions will take the following different forms:

(30) $$\left\{\begin{array}{l} \sigma_{l}=\frac{E^{+}(z)}{1-{\left(\mu^{+}\right)}^{2}}(\varepsilon_{l}+\mu^{+}\varepsilon_{\theta })\\ \sigma_{\theta }=\frac{E^{-}(z)}{1-{\left(\mu^{-}\right)}^{2}}(\varepsilon_{\theta }+\mu^{-}\varepsilon_{l}) \end{array}\right..$$

Substituting Equation (30) into Equation (28) and combining Equation (29), the strain energy of the neutral surface in the shell can be expressed as

(31) $$U_{\varepsilon 1}=\frac{1}{2[1-{\left(\mu^{+}\right)}^{2}]}\underset{V}{∭}E^{+}(z)\left[{\left(\varepsilon_{l}\right)}^{2}+\mu^{+}\varepsilon_{l}\varepsilon_{\theta }\right]dV+\frac{1}{2[1-{\left(\mu^{-}\right)}^{2}]}\underset{V}{∭}E^{-}(z)\left[{\left(\varepsilon_{\theta }\right)}^{2}+\mu^{-}\varepsilon_{l}\varepsilon_{\theta }\right]dV$$

Note that the relation between the angle d*φ* rotating around the cone top and its projection angle d*θ* on the vertical plane, *l*∙d*φ* = *l*∙sin*α*∙dθ (see Figure 2 and Figure 3), we have

(32) dφ=sinα⋅dθ,

thus, the differential volume element in Equation (31) d*V* = d*l*∙*l*d*φ*∙d*z* is now changed into d*V* = d*l* ∙ *l* ∙ sin*α* ∙ d*θ* ∙ d*z*, in which *l*_1_ < *l* < *l*_2_, 0 < *θ* < 2π and −*δ*_1_ < *z* < *δ*_2_. Due to the fact that in this problem, the displacements *w* and *u* are functions independent of *z* and *θ*, while the elastic moduli *E*^+^(*z*) and *E*^−^(*z*) are only functions of *z*, Equation (31) can be written as

(33) $$\begin{array}{cc} U_{\varepsilon 1} & =\frac{\pi \sin\alpha }{1-{\left(\mu^{+}\right)}^{2}}\int_{-\delta_{1}}^{\delta_{2}}E^{+}(z)dz\int_{l_{1}}^{l_{2}}\left[{\left(\varepsilon_{l}\right)}^{2}+\mu^{+}\varepsilon_{l}\varepsilon_{\theta }\right]ldl\\ & +\frac{\pi \sin\alpha }{1-{\left(\mu^{-}\right)}^{2}}\int_{-\delta_{1}}^{\delta_{2}}E^{-}(z)dz\int_{l_{1}}^{l_{2}}\left[{\left(\varepsilon_{\theta }\right)}^{2}+\mu^{-}\varepsilon_{l}\varepsilon_{\theta }\right]ldl \end{array}.$$

For the convenience of writing, we introduce the following coefficients

(34) $$D_{1}^{+}=\frac{1}{1-{\left(\mu^{+}\right)}^{2}}\int_{-\delta_{1}}^{\delta_{2}}E^{+}(z)dz=\frac{\delta }{\alpha_{1}}\frac{E_{0}e^{\alpha_{1}\delta_{2}/\delta }}{1-{\left(\mu^{+}\right)}^{2}}-\frac{\delta }{\alpha_{1}}\frac{E_{0}e^{-\alpha_{1}\delta_{1}/\delta }}{1-{\left(\mu^{+}\right)}^{2}},$$

(35) $$D_{1}^{-}=\frac{1}{1-{\left(\mu^{-}\right)}^{2}}\int_{-\delta_{1}}^{\delta_{2}}E^{-}(z)dz=\frac{\delta }{\alpha_{2}}\frac{E_{0}e^{\alpha_{2}\delta_{2}/\delta }}{1-{\left(\mu^{-}\right)}^{2}}-\frac{\delta }{\alpha_{2}}\frac{E_{0}e^{-\alpha_{2}\delta_{1}/\delta }}{1-{\left(\mu^{-}\right)}^{2}},$$

(36) $$D_{2}^{+}=\frac{1}{1-{\left(\mu^{+}\right)}^{2}}\int_{-\delta_{1}}^{0}E^{+}(z)z^{2}dz=\frac{E_{0}}{1-{\left(\mu^{+}\right)}^{2}}\left[\left(2\frac{\delta^{2}\delta_{1}}{{\alpha_{1}}^{2}}-\frac{\delta {\delta_{1}}^{2}}{\alpha_{1}}-2\frac{\delta^{3}}{{\alpha_{1}}^{3}}\right)e^{-\alpha_{1}\delta_{1}/\delta }\right.\left.+2{\left(\frac{\delta }{\alpha_{1}}\right)}^{3}\right],$$

(37) $$D_{2}^{-}=\frac{1}{1-{\left(\mu^{-}\right)}^{2}}\int_{0}^{\delta_{2}}E^{-}(z)z^{2}dz=\frac{E_{0}}{1-{\left(\mu^{-}\right)}^{2}}\left(\left[\frac{\delta {\delta_{2}}^{2}}{\alpha_{2}}-2\frac{\delta^{2}\delta_{2}}{{\alpha_{2}}^{2}}\right.+2\frac{\delta^{3}}{{\alpha_{2}}^{3}}\right)e^{\alpha_{2}\delta_{2}/\delta }\left.-2{\left(\frac{\delta }{\alpha_{2}}\right)}^{3}\right].$$

Substituting the first two equations of Equation (17) into Equation (33), the elastic strain energy *U_ε_*_1_ for the neutral surface deformation can be expressed as a function of displacement *w* and *u*, which is

(38) $$\begin{array}{cc} U_{\varepsilon 1} & =\pi D_{1}^{+}\sin\alpha \int_{l_{1}}^{l_{2}}\left\{\left[\frac{du}{dl}+\frac{1}{2}{\left(\frac{dw}{dl}\right)}^{2}\right]\left[l\frac{du}{dl}+\frac{l}{2}{\left(\frac{dw}{dl}\right)}^{2}+\mu^{+}\left(u+w\cot\alpha \right)\right]\right\}dl\\ & +\pi D_{1}^{-}\sin\alpha \int_{l_{1}}^{l_{2}}\left\{\left(u+w\cot\alpha \right)\left[\frac{u}{l}+\frac{w}{l}\cot\alpha +\mu^{-}\frac{du}{dl}+\mu^{-}\frac{1}{2}{\left(\frac{dw}{dl}\right)}^{2}\right]\right\}dl \end{array}.$$

Now let us derive the strain energy *U_ε_*_2_ of the bending deformation. Due to the introduction of a bimodular effect on materials, two states of tension and compression will occur simultaneously along the thickness direction. In order to consider this phenomenon, it is necessary to differentiate the tensile zone from the compression zone, and to calculate their corresponding strain energies separately. Therefore, the strain energy of bending deformation can be expressed as

(39) $$U_{\varepsilon 2}=\frac{1}{2}\underset{V^{-}}{∭}(\sigma_{l}^{-}\chi_{l}+\sigma_{\theta }^{-}\chi_{\theta }+\tau_{l\theta }^{-}\chi_{l\theta })zdV+\frac{1}{2}\underset{V^{+}}{∭}(\sigma_{l}^{+}\chi_{l}+\sigma_{\theta }^{+}\chi_{\theta }+\tau_{l\theta }^{+}\chi_{l\theta })zdV.$$

Because of the symmetry of the structure and load, we still have

(40) $$\tau_{l\theta }^{+}=\tau_{l\theta }^{-}=0,\;\chi_{l\theta }=0.$$

Note that $\sigma_{1}^{+}$, $\sigma_{1}^{-}$, $\sigma_{\theta }^{+}$, and $\sigma_{\theta }^{-}$ have already been given in Equation (9), substituting them into Equation (39) yields

(41) $$\begin{array}{cc} U_{\varepsilon 2} & =\frac{1}{2[1-{\left(\mu^{-}\right)}^{2}]}\underset{V^{-}}{∭}E^{-}(z)z^{2}\left(\chi_{l}\chi_{l}+2\mu^{-}\chi_{l}\chi_{\theta }+\chi_{\theta }\chi_{\theta }\right)dV\\ & +\frac{1}{2[1-{\left(\mu^{+}\right)}^{2}]}\underset{V^{+}}{∭}E^{+}(z)z^{2}\left(\chi_{l}\chi_{l}+2\mu^{+}\chi_{l}\chi_{\theta }+\chi_{\theta }\chi_{\theta }\right)dV \end{array}.$$

Since *w* and *u* are functions independent of *z* and *θ*, while the elastic moduli *E*^+^(*z*) and *E*^−^(*z*) are only functions of *z*, combining with Equations (32) and (41) can be further written as

(42) $$\begin{array}{ll} U_{\varepsilon 2} & =\frac{\pi \sin\alpha }{1-{\left(\mu^{-}\right)}^{2}}\int_{0}^{\delta_{2}}E^{-}(z)z^{2}dz\int_{l_{1}}^{l_{2}}\left(\chi_{l}\chi_{l}+2\mu^{-}\chi_{l}\chi_{\theta }+\chi_{\theta }\chi_{\theta }\right)ldl\\ & +\frac{\pi \sin\alpha }{1-{\left(\mu^{+}\right)}^{2}}\int_{-\delta_{1}}^{0}E^{+}(z)z^{2}dz\int_{l_{1}}^{l_{2}}\left(\chi_{l}\chi_{l}+2\mu^{+}\chi_{l}\chi_{\theta }+\chi_{\theta }\chi_{\theta }\right)ldl \end{array}.$$

Substituting the last two equations of Equation (17) into Equation (42) and considering Equations (36) and (37), we have

(43) $$\begin{array}{cc} U_{\varepsilon 2} & =\pi D_{2}^{-}\sin\alpha \int_{l_{1}}^{l_{2}}\left[{\left(\frac{d^{2}w}{dl^{2}}\right)}^{2}+2\mu^{-}\frac{1}{l}\frac{d^{2}w}{dl^{2}}\frac{dw}{dl}+{\left(\frac{1}{l}\frac{dw}{dl}\right)}^{2}\right]ldl\\ & +\pi D_{2}^{+}\sin\alpha \int_{l_{1}}^{l_{2}}\left[{\left(\frac{d^{2}w}{dl^{2}}\right)}^{2}+2\mu^{+}\frac{1}{l}\frac{d^{2}w}{dl^{2}}\frac{dw}{dl}+{\left(\frac{1}{l}\frac{dw}{dl}\right)}^{2}\right]ldl \end{array}.$$

### 4.3. Thermal Stress Work of Conical Shell W_T_

By analogy with the elastic strain energy of a structure under external mechanical loads, the thermal stress work can also be equivalent to the sum of the thermal strain energy *W_T_*_1_ for the neutral surface deformation and the thermal strain energy *W_T_*_2_ for the bending deformation, that is,

(44) $$W_{T}=W_{T1}+W_{T2}.$$

Since the thermal distribution along the thickness direction is non-uniform, the thermal strain energy of the bending deformation is not zero.

According to Equations (25) and (26), and the assumption of neglecting normal strain $\varepsilon_{z}$, the thermal stress work caused by the neutral surface deformation can be expressed as

(45) $$W_{T1}=\underset{V}{∭}\sigma_{t}(\varepsilon_{l}+\varepsilon_{\theta })dV,$$

where the thermal stress *σ*_*t*_ has been given in Equation (2). Combining Equation (2) and the first two equations of Equation (17), Equation (45) may be written as

(46) $$W_{T1}=\frac{1}{1-\mu^{-}}{∭}_{V}E^{-}(z)\alpha (z)\left[T(z)-T_{0}\right]\left[\frac{du}{dl}+\frac{1}{2}{\left(\frac{dw}{dl}\right)}^{2}+\frac{u}{l}+\frac{w}{l}\cot\alpha \right]dV.$$

This study only considers the non-uniform thermal distribution along the thickness direction, so the thermal stress is only a function of *z*. Obviously, *E*^−^(*z*), *α*(*z*), and *T*(*z*) are only functions of *z*, and the displacements *w* and *u* are only related to *l*. First, we calculate the integral of thermal stress along the thickness direction, which is called the thermal membrane force *N_T_*, and is expressed as

(47) $$N_{T}=\int_{-\delta_{1}}^{\delta_{2}}\sigma_{t}dz=\int_{-\delta_{1}}^{\delta_{2}}\frac{E^{-}(z)}{1-\mu^{-}}\alpha (z)\left[T(z)-T_{0}\right]dz.$$

Note that again d*V* = d*l* ∙ *l* ∙ sin*α* ∙ d*θ* ∙ d*z*, Equation (46) is transformed into a cubic integral

(48) $$W_{T1}=\sin\alpha \int_{0}^{2\pi }d\theta \int_{-\delta_{1}}^{\delta_{2}}\sigma_{t}dz\int_{l_{1}}^{l_{2}}\left[l\frac{du}{dl}+\frac{1}{2}{\left(\frac{dw}{dl}\right)}^{2}+u+w\cdot \cot\alpha \right]dl.$$

Combining Equation (47), we have

(49) $$W_{T1}=2\pi N_{T}\sin\alpha \int_{l_{1}}^{l_{2}}\left[l\frac{du}{dl}+\frac{1}{2}l{\left(\frac{dw}{dl}\right)}^{2}+u+w\cdot \cot\alpha \right]dl.$$

According to Equation (26) and the assumption of neglecting normal strain $\varepsilon_{z}$, the thermal stress work *W_T_*_2_ caused by bending deformation can be expressed as

(50) $$W_{T2}=\underset{V}{∭}\sigma_{t}z(\chi_{l}+\chi_{\theta })dV.$$

The thermal stress *σ_t_* is only a function of *z*, and the displacements *w* and *u* are only related to *l*. Similarly, we may convert the triple integral in Equation (50) into a cubic integral

(51) $$W_{T2}=\sin\alpha \int_{0}^{2\pi }d\theta \int_{-\delta_{1}}^{\delta_{2}}\sigma_{t}zdz\int_{l_{1}}^{l_{2}}(\chi_{l}+\chi_{\theta })ldl.$$

Since $\sigma_{t}$ represents the thermal stress at a certain point, and the integral of the term *σ_t_* represents the bending moment generated by non-uniform thermal distribution along the thickness direction *z*, which is called thermal bending moment *M_T_*, and is expressed as

(52) $$M_{T}=\int_{-\delta_{1}}^{\delta_{2}}\sigma_{t}zdz=\int_{-\delta_{1}}^{\delta_{2}}\frac{E^{-}(z)}{1-\mu^{-}}\alpha (z)\left[T(z)-T_{0}\right]zdz.$$

Substituting the last two equations of Equation (17) into Equation (51) and considering Equation (52) yields

(53) $$W_{T2}=2\pi M_{T}\sin\alpha \int_{l_{1}}^{l_{2}}\left(-l\frac{d^{2}w}{dl^{2}}-\frac{dw}{dl}\right)dl.$$

At this point, both the strain energy *U_ε_* and thermal stress work *W_T_* of the bimodular functionally graded truncated conical shell under mechanical and thermal loads have been expressed in terms of displacement *w* and *u*.

### 4.4. Ritz Method

#### 4.4.1. Displacement Function and Variation Equation

The constraints of truncated conical shells considered in this study include the following two types: one is fixed at both ends and another is simply supported at both ends. The corresponding boundary conditions will give, for the fixed shell, *u* = *w* = d*w*/d*l* = 0, at *l* = *l*_1_ and *l*_2_; while for simply supported shell, *u* = *w* = d^2^*w*/d*l*^2^ = 0, at *l* = *l*_1_ and *l*_2_. For the fixed shell, we take the following displacement functions:

(54) $$\left\{\begin{array}{l} u=\sum_{n=0}^{\infty }A_{n}u_{n}=(l-l_{1})(l-l_{2})\left[\kappa_{0}+\kappa_{1}(l-l_{1})+\kappa_{2}{\left(l-l_{1}\right)}^{2}+\cdots \right]\\ w=\sum_{m=0}^{\infty }B_{m}w_{m}=(l-l_{1}{)}^{2}(l-l_{2}{)}^{2}\left[\lambda_{0}+\lambda_{1}(l-l_{1})+\lambda_{2}{\left(l-l_{1}\right)}^{2}+\cdots \right] \end{array}\right.,$$

which satisfy all boundary conditions, in which *κ_i_* and *λ_i_* (*i* = 0, 1, 2 …) are undetermined coefficients.

Note that for simply supported shell, d^2^*w*/d*l*^2^ = 0 at *l* = *l*_1_ and *l*_2_ should be satisfied, which makes it not so easy to construct the displacement pattern. For this purpose, we first suppose a function *g*(*l*) as follows:

(55) $$g(l)=(l-l_{1})(l-l_{2}),$$

which satisfies *g*(*l*) = 0 at *l* = *l*_1_ and *l*_2_. Doubly integrating this function with respect to *l*, we have another function *f* (*l*), which gives

(56) $$f(l)=\frac{1}{12}l^{4}-\frac{1}{6}l^{3}l_{1}-\frac{1}{6}l^{3}l_{2}+\frac{1}{2}l_{1}l_{2}l^{2}+C_{1}l+C_{2},$$

where *C*_1_ and *C*_2_ are two integration constants, which may be determined as follows: we let the function *f* (*l*) be a factor in the displacement *w*, and thus, *f* (*l*) should be zero *l* = *l*_1_ and *l*_2_, which gives

(57) $$\left\{\begin{array}{l} f(l_{1})=\frac{1}{12}{l_{1}}^{4}-\frac{1}{6}{l_{1}}^{3}l_{1}-\frac{1}{6}{l_{1}}^{3}l_{2}+\frac{1}{2}l_{2}{l_{1}}^{3}+C_{1}l_{1}+C_{2}=0\\ f(l_{2})=\frac{1}{12}{l_{2}}^{4}-\frac{1}{6}{l_{2}}^{3}l_{1}-\frac{1}{6}{l_{2}}^{3}l_{2}+\frac{1}{2}l_{1}{l_{2}}^{3}+C_{1}l_{2}+C_{2}=0 \end{array}\right.,$$

thus, *C*_1_ and *C*_2_ may be determined as

(58) $$\left\{\begin{array}{l} C_{1}=\frac{1}{12}(l_{1}^{3}+l_{2}^{3})-\frac{1}{4}(l_{2}l_{1}^{2}+l_{2}^{2}l_{1})\\ C_{2}=-\frac{1}{12}(l_{2}^{3}l_{1}+l_{1}^{3}l_{2})+\frac{1}{4}l_{2}^{2}l_{1}^{2} \end{array}\right..$$

Replacing the *C*_1_ and *C*_2_ in Equation (56), we have

(59) $$f(l)=\frac{1}{12}l^{4}-\frac{1}{6}(l_{1}+l_{2})l^{3}+\frac{1}{2}l_{1}l_{2}l^{2}+\left(\frac{1}{12}l_{1}^{3}-\frac{1}{4}l_{2}l_{1}^{2}-\frac{1}{4}l_{2}^{2}l_{1}+\frac{1}{12}l_{2}^{3}\right)l-\frac{1}{12}l_{2}^{3}l_{1}-\frac{1}{12}l_{1}^{3}l_{2}+\frac{1}{4}l_{2}^{2}l_{1}^{2}.$$

Finally, the displacement function under a simply supported constraint may be prescribed as

(60) $$\left\{\begin{array}{l} u=\sum_{n=0}^{\infty }A_{n}u_{n}=(l-l_{1})(l-l_{2})\left[\upsilon_{0}+\upsilon_{1}(l-l_{1})+\upsilon_{2}{\left(l-l_{1}\right)}^{2}+\cdots \right]\\ w=\sum_{m=0}^{\infty }B_{m}w_{m}=f(l)\left[\varsigma_{0}+\varsigma_{1}(l-l_{1})+\varsigma_{2}{\left(l-l_{1}\right)}^{2}+\cdots \right] \end{array}\right.,$$

which satisfies all simply supported boundary conditions, in which *υ_i_* and *ς_i_* (*i* = 0, 1, 2 …) are undetermined coefficients.

Referring to Equations (54) and (60), the variations of displacement components *w* and *u* are

(61) $$\left\{\begin{array}{l} \delta u=\sum_{n=0}^{\infty }u_{n}\delta A_{n}\\ \delta w=\sum_{m=0}^{\infty }w_{m}\delta B_{m} \end{array}\right..$$

When only thermal load is applied, according to the principle of minimum potential energy, the relation between the strain energy *U_ε_* of the shell and the work conducted by thermal stress *W_T_* is

(62) $$\delta (U_{\varepsilon }-W_{T})=0,$$

the corresponding variation of strain energy and thermal stress work is

(63) $$\delta (U_{\varepsilon }-W_{T})=\sum_{n,m=0}^{\infty }\left[\frac{\partial (U_{\varepsilon }-W_{T})}{\partial A_{n}}\delta A_{n}+\frac{\partial (U_{\varepsilon }-W_{T})}{\partial B_{m}}\delta B_{m}\right].$$

When thermal load and external force are applied simultaneously, according to the principle of energy conservation, the increase in structural strain energy should be equal to the work conducted by the external force. Therefore, the relation between the structural strain energy, thermal stress work, and external force work is obtained as follows:

(64) $$\delta (U_{\varepsilon }-W_{T})=\int_{V}(f_{l}\delta u+f_{z}\delta w)dV+\int_{S}({\bar{f}}_{l}\delta u+{\bar{f}}_{z}\delta w)dS,$$

where $f_{l}$ and $f_{z}$ represent the body force along the direction of the generatrix *l* and the normal *z*, respectively, $\bar{f_{l}}$ and $\bar{f_{z}}$ represents the surface forces along the two directions, respectively. Combining Equations (61), (63) and (64), we have

(65) $$\begin{array}{l} \sum_{n,m=0}^{\infty }\left[\frac{\partial (U_{\varepsilon }-W_{T})}{\partial A_{n}}\delta A_{n}+\frac{\partial (U_{\varepsilon }-W_{T})}{\partial B_{m}}\delta B_{m}\right]\\ =\sum_{n,m=0}^{\infty }\left(\int_{V}f_{l}u_{n}\delta A_{n}dV+\int_{V}f_{w}w_{m}\delta B_{m}dV\right)+\sum_{n,m=0}^{\infty }\left(\int_{S}{\bar{f}}_{l}u_{n}\delta A_{n}dS+\int_{S}{\bar{f}}_{w}w_{m}\delta B_{m}dS\right) \end{array}.$$

After simplification, we obtain

(66) $$\begin{array}{l} \sum_{n=0}^{\infty }\left[\frac{\partial (U_{\varepsilon }-W_{T})}{\partial A_{n}}-\int_{V}f_{l}u_{n}dV-\int_{S}{\bar{f}}_{l}u_{n}dS\right]\delta A_{n}\\ +\sum_{m=0}^{\infty }\left[\frac{\partial (U_{\varepsilon }-W_{T})}{\partial B_{m}}-\int_{V}f_{w}w_{m}dV-\int_{S}{\bar{f}}_{w}w_{m}dS\right]\delta B_{m}=0 \end{array}.$$

Since the assumptions of the variations δ*A_n_* and δ*B_n_* are arbitrary, to satisfy the equation above, the corresponding coefficients of the variations δ*A_n_* and δ*B_n_* must be zero, namely,

(67) $$\left\{\begin{array}{l} \frac{\partial (U_{\varepsilon }-W_{T})}{\partial A_{n}}-\int_{V}f_{l}u_{n}dV-\int_{S}{\bar{f}}_{l}u_{n}dS=0\\ \frac{\partial (U_{\varepsilon }-W_{T})}{\partial B_{m}}-\int_{V}f_{w}w_{m}dV-\int_{S}{\bar{f}}_{w}w_{m}dS=0 \end{array}\right.,$$

which may be used for the solution of displacement components *u* and *w*. As long as *n* and *m* are sufficiently large, the solutions of these unknown variables will approach their true values. Next, we will solve the problem by distinguishing the boundary cases.

#### 4.4.2. Conical Shell with Two Ends Fixed

To simplify the calculation and show briefly the solution process, only the first few terms of the series in Equation (54) are used for calculation. Specifically, if *κ*_0_, *κ*_1_ and *λ*_0_ in Equation (54) are used; substituting it into Equations (27) and (44), we will have the strain energy *U_f_* and thermal stress work *W_f_* as follows:

(68) $$\begin{array}{cc} U_{f} & =U_{\varepsilon 1}+U_{\varepsilon 2}\\ & =f_{1}\kappa_{0}\kappa_{1}+f_{2}\kappa_{0}{\lambda_{0}}^{2}+f_{3}{\kappa_{0}}^{2}+f_{4}\kappa_{1}\lambda_{0}+f_{5}\kappa_{1}{\lambda_{0}}^{2}+f_{6}{\kappa_{1}}^{2}+f_{7}{\lambda_{0}}^{4}+f_{8}{\lambda_{0}}^{3}+f_{9}\kappa_{0}\lambda_{0}+f_{10}{\lambda_{0}}^{2} \end{array},$$

(69) $$W_{f}=W_{T1}+W_{T2}=g_{1}\lambda_{0}+g_{2}{\lambda_{0}}^{2},$$

where the coefficients *f*_1_~*f*_10_, *g*_1_ and $g_{2}$ are shown in Appendix A.

In our study, the influence of body force of the shell is neglected, thus there exists only the surface force *q* acting on the shell. Thus, Equation (67) is simplified as (note that d*S* = d*l* ∙ *l* ∙ sin*α* ∙ d*θ*)

(70) $$\left\{\begin{array}{l} \frac{\partial (U_{f}-W_{f})}{\partial A_{n}}=0\\ \frac{\partial (U_{f}-W_{f})}{\partial B_{m}}=2\pi \sin\alpha \int_{l_{1}}^{l_{2}}qw_{m}ldl \end{array}\right..$$

The right-hand side of the second equation in Equation (70) is evidently associated solely with the load *q*, and can be expressed as

(71) $$q_{1}=2\pi \sin\alpha \int_{l_{1}}^{l_{2}}qw_{0}ldl,$$

where the expression of *q*_1_ can be found in the Appendix A. Substituting Equations (68) and (69) into Equation (70), and subsequently differentiating with respect to *κ*_0_, *κ*_1_, and *λ*_0_, we have

(72) $$\left\{\begin{array}{l} f_{2}\lambda_{0}^{2}+f_{1}\kappa_{1}+2f_{3}\kappa_{0}+f_{9}\lambda_{0}=0\\ f_{5}\lambda_{0}^{2}+f_{1}\kappa_{0}+f_{4}\lambda_{0}+2f_{6}\kappa_{1}=0 \end{array}\right.,$$

(73) $$4f_{7}\lambda_{0}^{3}+2f_{2}\kappa_{0}\lambda_{0}+2f_{5}\kappa_{1}\lambda_{0}+3f_{8}\lambda_{0}^{2}+2f_{10}\lambda_{0}+f_{4}\kappa_{1}+f_{9}\kappa_{0}-2g_{2}\lambda_{0}-g_{1}=q_{1}.$$

Solving Equation (72), the *κ*_0_ and *κ*_1_ may be expressed in terms of *λ*_0_ as follows

(74) $$\kappa_{0}=\frac{2f_{6}f_{9}-f_{1}f_{4}}{f_{1}^{2}-4f_{3}f_{6}}\lambda_{0}+\frac{2f_{2}f_{6}-f_{1}f_{5}}{f_{1}^{2}-4f_{3}f_{6}}{\lambda_{0}}^{2},$$

(75) $$\kappa_{1}=\frac{2f_{3}f_{4}-f_{1}f_{9}}{f_{1}^{2}-4f_{3}f_{6}}\lambda_{0}+\frac{2f_{3}f_{5}-f_{1}f_{2}}{f_{1}^{2}-4f_{3}f_{6}}{\lambda_{0}}^{2}.$$

Substituting Equations (74) and (75) into Equation (73), we have

(76) $$\xi_{3}\lambda_{0}^{3}+\xi_{2}\lambda_{0}^{2}+\xi_{1}\lambda_{0}+\xi_{0}=0,$$

where the coefficients *ξ*_0_~*ξ*_3_ are shown in Appendix A. Finally, the *λ*_0_ may be solved as

(77) $$\lambda_{0}=\frac{1}{6\xi_{3}}\sqrt[{3}]{\vartheta_{1}}-\frac{2}{3\xi_{3}\sqrt[{3}]{\vartheta_{1}}}\left(3\xi_{1}\xi_{3}-\xi_{2}^{2}\right)-\frac{\xi_{2}}{3\xi_{3}},$$

where

(78) $$\vartheta_{1}=12\sqrt{3}\sqrt{27\xi_{0}^{2}\xi_{3}^{2}-18\xi_{0}\xi_{1}\xi_{2}\xi_{3}+4\xi_{0}\xi_{2}^{3}+4\xi_{1}^{3}\xi_{3}-\xi_{1}^{2}\xi_{2}^{2}\xi_{3}}-108\xi_{0}\xi_{3}^{2}+36\xi_{1}\xi_{2}\xi_{3}-8\xi_{2}^{3}$$

#### 4.4.3. Conical Shell with Two Ends Simply Supported

If *υ*_0_, *υ*_1_, and *ς*_0_ in Equation (60) are used, substituting it into Equations (27) and (44), we will have the strain energy *U_h_* and thermal stress work *W_h_* as follows:

(79) $$\begin{array}{l} U_{h}=U_{\varepsilon 1}+U_{\varepsilon 2}\\ =h_{1}\upsilon_{0}{\varsigma_{0}}^{2}+h_{2}\upsilon_{0}\upsilon_{1}+h_{3}{\upsilon_{0}}^{2}+h_{4}\upsilon_{1}\varsigma_{0}+h_{5}\upsilon_{1}{\varsigma_{0}}^{2}+h_{6}{\upsilon_{1}}^{2}+h_{7}{\varsigma_{0}}^{3}+h_{8}{\varsigma_{0}}^{4}+h_{9}\upsilon_{0}\varsigma_{0}+h_{10}{\varsigma_{0}}^{2}+h_{11}\varsigma_{0} \end{array},$$

(80) $$W_{h}=W_{T1}+W_{T2}=p_{1}\varsigma_{0}+p_{2}{\varsigma_{0}}^{2},$$

where the coefficients $h_{1}$~*h*_11_, $p_{1}$ and *p*_2_ are shown in Appendix B. Substituting Equations (79) and (80) into Equation (70), and subsequently differentiating with respect to *υ*_0_, *υ*_1_, and *ς*_0_, we have

(81) $$\left\{\begin{array}{l} h_{1}\varsigma_{0}^{2}+h_{2}\upsilon_{1}+2h_{3}\upsilon_{0}+h_{9}\varsigma_{0}-p_{2}=0\\ h_{5}\varsigma_{0}^{2}+h_{2}\upsilon_{0}+h_{4}\varsigma_{0}+2h_{6}\upsilon_{1}-p_{4}=0 \end{array}\right.,$$

(82) $$4h_{8}\varsigma_{0}^{3}+2h_{1}\upsilon_{0}\varsigma_{0}+2h_{5}\upsilon_{1}\varsigma_{0}+3h_{7}\varsigma_{0}^{2}+2h_{10}\varsigma_{0}+h_{4}\upsilon_{1}+h_{9}\upsilon_{0}-2p_{3}\varsigma_{0}+h_{11}-p_{1}=q_{2},$$

where $q_{2}$ is the same as that of Equation (71), with only difference in *w*_0_, and the expression of *q*_2_ can be found in the Appendix B. Solving Equation (81), the *υ*_0_ and *υ*_1_ may be expressed in terms of *ς*_0_ as follows:

(83) $$\upsilon_{0}=\frac{2h_{1}h_{6}\varsigma_{0}^{2}-h_{2}h_{5}\varsigma_{0}^{2}-h_{2}h_{4}\varsigma_{0}+2h_{6}h_{9}\varsigma_{0}+h_{2}p_{4}-2h_{6}p_{2}}{h_{2}^{2}-4h_{3}h_{6}},$$

(84) $$\upsilon_{1}=-\frac{h_{1}h_{2}\varsigma_{0}^{2}-2h_{3}h_{5}\varsigma_{0}^{2}+h_{2}h_{9}\varsigma_{0}-2h_{3}h_{4}\varsigma_{0}-h_{2}p_{2}+2h_{3}p_{4}}{h_{2}^{2}-4h_{3}h_{6}}.$$

Substituting Equations (83) and (84) into Equation (82), we have

(85) $$\eta_{3}{\varsigma_{0}}^{3}+\eta_{2}{\varsigma_{0}}^{2}+\eta_{1}\varsigma_{0}+\eta_{0}=0,$$

where the coefficients $\eta_{0}$~*η*_3_ are shown in Appendix B. Finally, the *ς*_0_ may be solved as

(86) $$\varsigma_{0}=\frac{1}{6\eta_{3}}\sqrt[{3}]{\vartheta_{2}}-\frac{2}{3\eta_{3}\sqrt[{3}]{\vartheta_{2}}}\left(3\eta_{1}\eta_{3}-{\eta_{2}}^{2}\right)-\frac{\eta_{2}}{3\eta_{3}},$$

where

(87) $$\vartheta_{2}=12\sqrt{3}\sqrt{27\eta_{0}^{2}\eta_{3}^{2}-18\eta_{0}\eta_{1}\eta_{2}\eta_{3}+4\eta_{0}\eta_{2}^{3}+4\eta_{1}^{3}\eta_{3}-\eta_{1}^{2}\eta_{2}^{2}\eta_{3}}-108\eta_{0}\eta_{3}^{2}+36\eta_{1}\eta_{2}\eta_{3}-8\eta_{2}^{3}$$

## 5. Examples and Numerical Simulation

In this section, we will conduct numerical simulation and compare the numerical results with the variation solution obtained in Section 4.

### 5.1. Computational Examples

The elastic modulus of the bimodular FGMs is discussed in Section 2. By appropriately selecting the gradient indices *α*_1_ and *α*_2_ in the calculation, the bimodular effect on the functionally graded material can be accurately characterized. Based on our previous study [14], four groups of material data with different gradient indices are selected, as shown in Table 1, which corresponds to the four cases of bimodular effect, respectively. The remaining material parameters are listed in Table 2, in which the heat-related parameters are selected by referring to [5].

There are two types of loads considered in this paper, namely, the external pressure (mechanical load) and the temperature rise (thermal load). In addition, the boundary cases with two ends fully fixed and simply supported are also considered. Some geometric and load parameters of the truncated conical shell are listed in Table 3. In addition, according to the derivation of the neutral layer and the above parameters shown in Table 1, Table 2 and Table 3, we can first calculate the tensile and compressive thicknesses, *δ*_1_ and *δ*_2_, which are listed in Table 4.

With the data in Table 1, Table 2, Table 3 and Table 4, the specific deformation values of the conical shell under different constraints and load types can be obtained, which may be used for the comparison with the results of numerical simulation.

### 5.2. Numerical Simulation

In this section, we will perform the numerical simulation and compare the results with those obtained using the variation method. The current commercial finite element software does not include a pre-existing constitutive model for bimodular functionally graded material. For this purpose, a subroutine for this material in Abaqus/CAE 2022, that is, user materials subroutine (UMAT), has been developed in our previous study [14]. This study will build upon this foundation to enhance the subroutine’s ability to accurately represent thermal responses.

For bimodular characteristics, we still follow the principal stress discrimination method proposed by Ambartsumyan, that is, the elastic modulus of a certain point is selected according to the principal stress state of this point. The calculation steps of the program are as follows:

(i) The material parameters are received from the Abaqus main program at the first increment, and the elastic modulus of bimodular FGMs along the thickness direction is defined in the tensile and compressive states, respectively. Because of the geometric nonlinearity, the elastic modulus of bimodular FGMs dependent on initial coordinates can only be defined correctly in the first increment step;

(ii) Based on the classical Jacobi method, the principal stresses and principal directions will be obtained by calculating the eigenvalues and eigenvectors of the stress matrix;

(iii) According to the direction of principal stress obtained, the due constitutive relationship of each element is determined, and the stiffness matrix of each element is collected to form the total stiffness matrix

(88) $$D=\left(\begin{array}{cccccc} a_{11} & a_{12} & a_{13} & 0 & 0 & 0\\ a_{21} & a_{22} & a_{23} & 0 & 0 & 0\\ a_{31} & a_{32} & a_{33} & 0 & 0 & 0\\ 0 & 0 & 0 & G & 0 & 0\\ 0 & 0 & 0 & 0 & G & 0\\ 0 & 0 & 0 & 0 & 0 & G \end{array}\right),$$

where *a_ij_* and the equivalent shear modulus *G* are

(89) $$a_{ij}=\left\{\begin{array}{l} \frac{E_{i}}{\mu_{1}\mu_{2}+\mu_{1}\mu_{3}+\mu_{2}\mu_{2}+2\mu_{1}\mu_{2}\mu_{3}-1}\left(\frac{1}{\mu_{i}}\prod_{k=1}^{3}\mu_{k}-1\right)\;i=j\\ \frac{-E_{i}}{\mu_{1}\mu_{2}+\mu_{1}\mu_{3}+\mu_{2}\mu_{2}+2\mu_{1}\mu_{2}\mu_{3}-1}\left(\frac{1}{\mu_{i}}\prod_{k=1}^{3}\mu_{k}-\mu_{j}\right)\;i\neq j \end{array}\right.\;i,j=1,2,3,$$

(90) $$G=\frac{\eta E^{+}+\left(1-\eta \right)E^{-}}{2\eta \left(1+\mu^{+}\right)+2\left(1-\eta \right)\left(1+\mu^{-}\right)},$$

where *E_i_* and *μ_i_* are the elastic modulus and Poisson’s ratio in the direction *i*, which are determined by the calculated principal stress direction; *η* is the accelerated convergence factor whose value is the ratio of the sum of the tensile principal stresses to the sum of absolute values of all principal stresses;

(iv) In this increment step, Abaqus is able to input a strain increment to calculate a stress increment and update the stress matrix, that is,

(91) $$\sigma_{t+1}=\sigma_{t+1}+d\sigma =\sigma_{t+1}+Dd\varepsilon^{e}=D\left(d\varepsilon -d\varepsilon^{t}\right),$$

where d*ε^e^* is the elastic strain increment, and d*ε^t^* is the thermal strain increment. If the equilibrium condition is reached based on the stress matrix *σ*^t+1^, the next increment step will be implemented; otherwise, a lesser strain increment Δ*ε* is able to be re-input until the equilibrium condition is satisfied.

**Figure 4:**
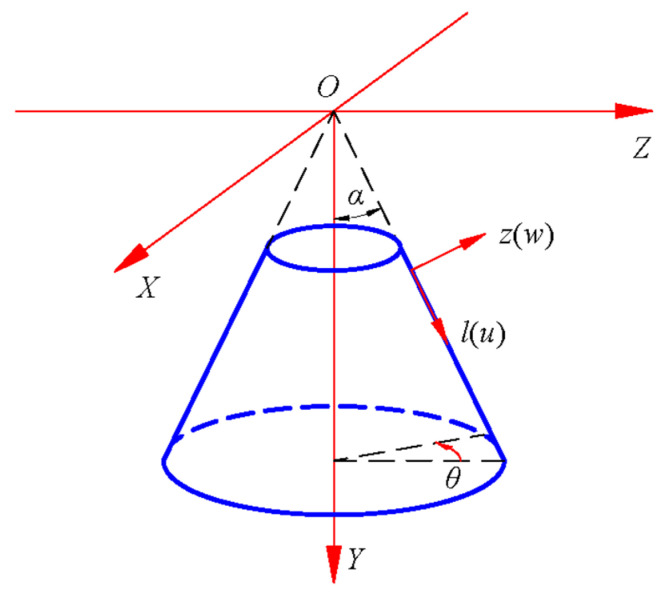
Orthogonal curvilinear coordinates and global coordinates.

Based on the parameters in Table 1, Table 2, Table 3 and Table 4, four groups of gradient indices, two kinds of boundary constraints, and two types of loads are selected for numerical simulation. When constructing the material subroutine, it is important to note that the previous derivation was established on orthogonal curvilinear coordinates on the neutral surface, while Abaqus employs global coordinates. Therefore, a coordinate system transformation is necessary when defining material properties that vary along the thickness direction. As shown in Figure 4, the relation between the two coordinate systems is

(92) $$\left\{\begin{array}{l} Y=l\cos\alpha -z\sin\alpha \\ X=\left(l\sin\alpha +z\cos\alpha \right)\cos\theta \\ Z=\left(l\sin\alpha +z\cos\alpha \right)\sin\theta \end{array}\right..$$

In the UMAT subroutine, the *X*, *Y*, and *Z* axes are represented by COORDS(1), COORDS(2), and COORDS(3), respectively. Obviously, the coordinate *z* in Figure 4 is consistent with the relative distance between the integral point and the neutral surface; therefore, according to the above formula, *z* is expressed as

(93) $$\begin{array}{l} z=\sqrt{X^{2}+Z^{2}}\cos\alpha -Y\sin\alpha \\ =\sqrt{{\left(\mathrm{COORDS}(1)\right)}^{2}+{\left(\mathrm{COORDS}(3)\right)}^{2}}\cos\alpha -\mathrm{COORDS}(2)\sin\alpha \end{array}.$$

**Figure 5:**
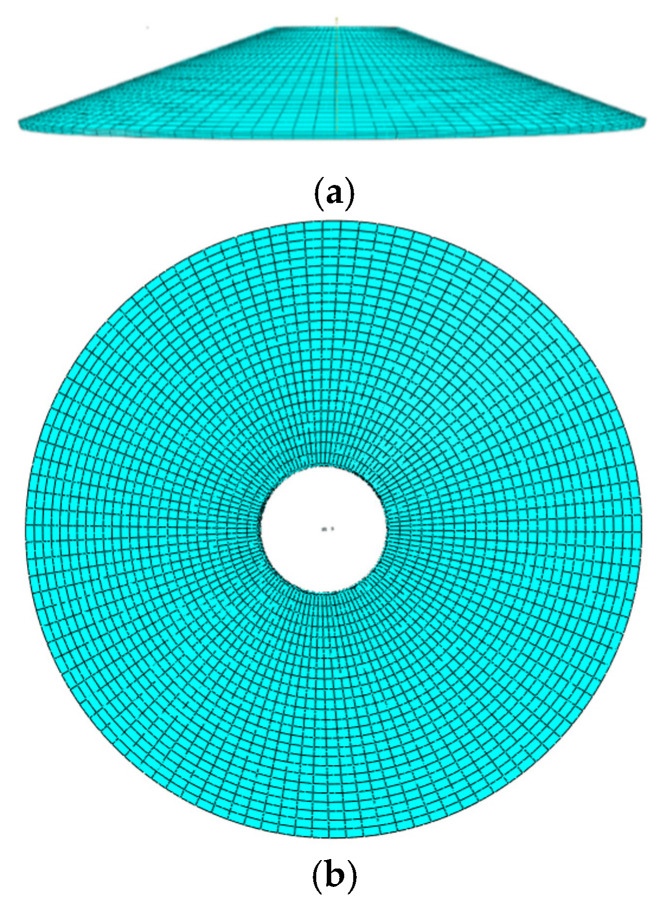
Truncated conical shell model. (**a**) Elevation and (**b**) plan.

During the mesh division, the mesh size on the neutral surface is 0.2m, and the shell is divided into 3 layers along the thickness direction. The mesh division is completed in a mapped way, and thus, a total of 5280 elements are generated. C3D20 (quadratic-order three-dimensional solid element with twenty nodes) is adopted as the element type. Note that to simulate gradient change along thickness direction of FGMs, we use full 3D models but not a shell axisymmetric model. Figure 5 shows the elevation and plan of the truncated conical shell model in numerical simulation. The “Static, General” is able to be used as the analysis method in the analysis step section; the initial and maximum increments are set to 0.01 and 0.1, respectively.

**Figure 6:**
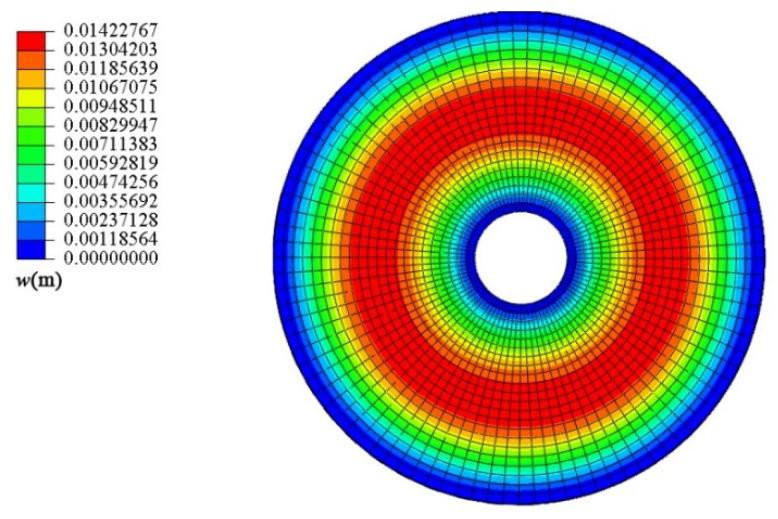
Displacement contour plot of conical shell with two ends fixed (*T* = 120 °C).

**Figure 7:**
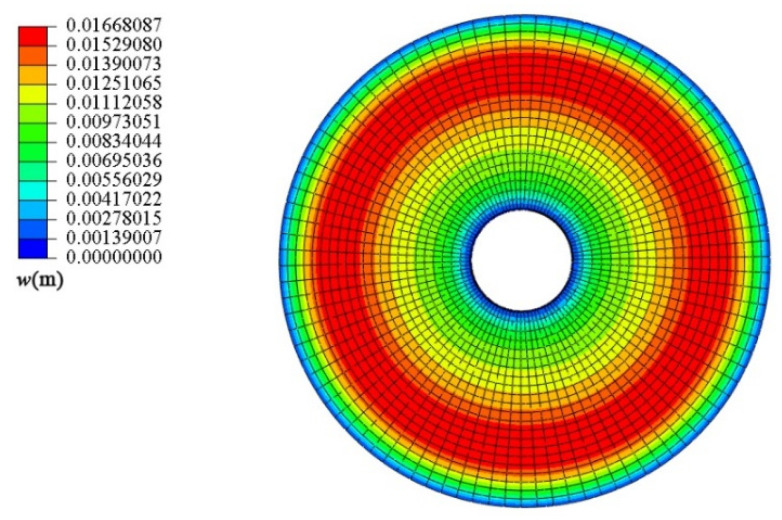
Displacement contour plot of conical shell with two ends simply supported (*T* = 120 °C).

**Figure 8:**
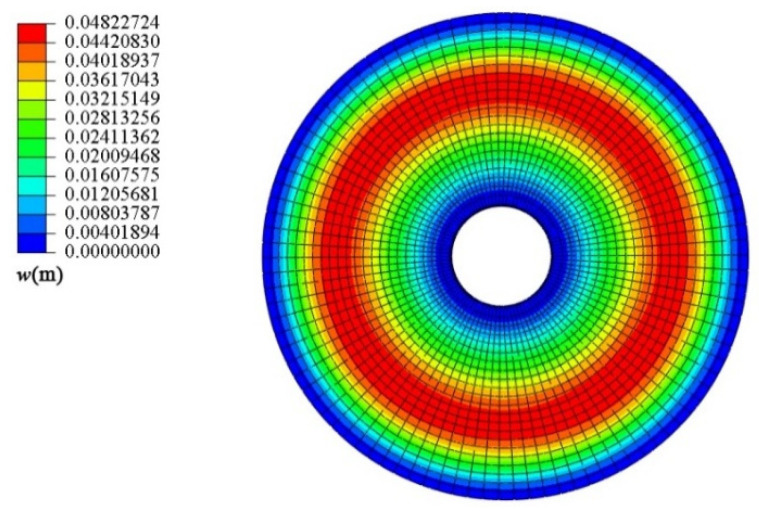
Displacement contour plot of conical shell with two ends fixed (*q* = 1.0 MPa).

**Figure 9:**
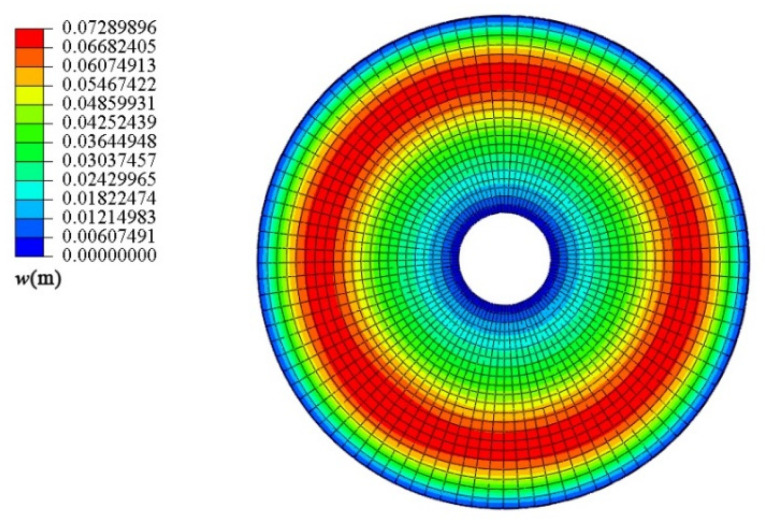
Displacement contour plot of conical shell with two ends simply supported (*q* = 1.0 MPa).

Next, the conical shell under two kinds of boundary constraints and two types of loads is numerically simulated, respectively, and the ratio of the maximum deflection to thickness of the conical shell is defined as *W*_max_, which is dimensionless. Figure 6, Figure 7, Figure 8 and Figure 9 show the displacement contour plot of the truncated conical shell with two ends fixed and simply supported, and under the thermal and mechanical loads, respectively.

**Figure 10:**
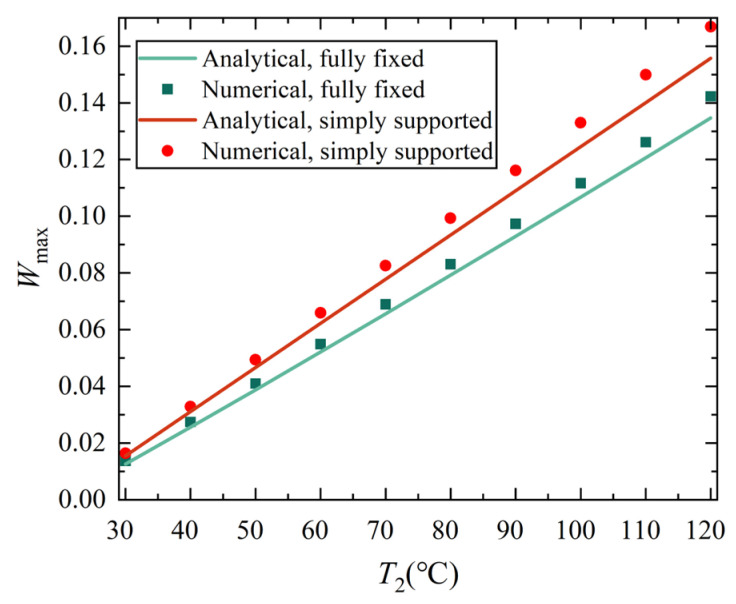
The maximum deflection variation curve with increasing temperature of analytical solutions and numerical results of conical shell under two boundary conditions.

**Figure 11:**
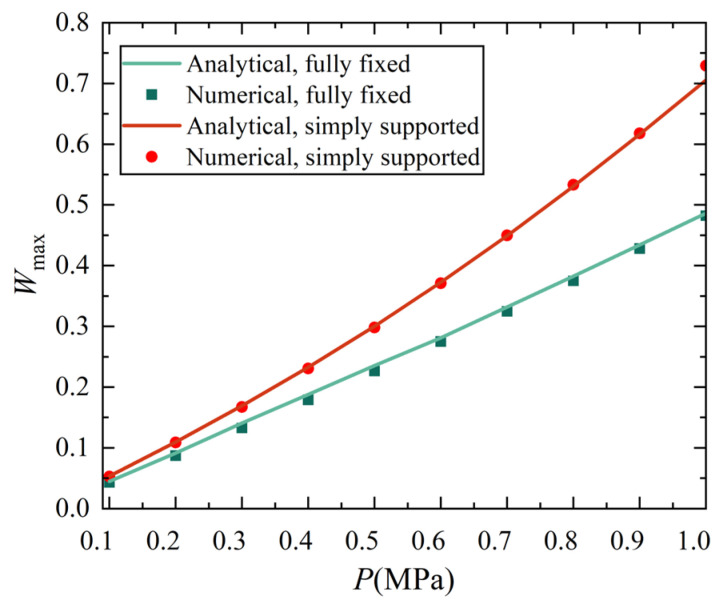
The maximum deflection variation curve with increasing transverse mechanical load of analytical solutions and numerical results of conical shell under two boundary conditions.

Figure 10 and Figure 11 show the variation curves of the maximum deflection varying with thermal and mechanical loads, also indicating that the discrepancies between variation solutions and FE simulation results are acceptably small. The maximum deflection values of two solutions are given in Table A1 and Table A2 in Appendix C for reference. From Figure 10 and Figure 11, it is easy to see that discrepancies still exist between the numerical simulation and variation solution, which may stem from differences in mechanical models utilized, certain assumptions adopted during analytical solving, or insufficient terms accounted for in the displacement function. Nevertheless, these discrepancies fall within an acceptable range, affirming the validity of the variation solution. It is also observed from Figure 10 and Figure 11 that the nonlinear characteristic is not very obvious, especially for Figure 10, which shows an almost linear relationship. The reason for this phenomenon may be that the dimensionless maximum deflection *W*_max_ (that is, the ratio of real deflection to shell thickness) is less than 1/5, and does not fall into the range of the large deflection of plates and shells which is generally taken as 1/5 < *W*_max_ < 5.

In addition, it is easy to find that although the maximum displacement W_max_ of the conical shell with two ends fixed is slightly smaller than that of the conical shell with two ends simply supported under both thermal load and mechanical load, there is no significant difference between the two different boundary constraints, especially under thermal load. However, by observing the displacement contour plot, it is easy to see that there is a significant difference in the position of the maximum displacement point of the conical shell with two ends fixed or simply supported, which will be analyzed in detail in the next section.

## 6. Results and Discussion

Since the validity of the variation solution has been verified, in this section, we will apply this solution to the discussion and parameter study.

### 6.1. Bimodular Functionally Graded Effect on Deflection

For the convenience of comparison, we introduce the following dimensionless quantities:

(94) $$\xi =\frac{l-l_{1}}{l_{2}-l_{1}},$$

(95) $$W=\frac{\left|w\right|}{\delta },$$

to stand for the location coordinate (0 < *ξ* < 1) and deflection (*W* > 0), respectively, in which, for the deflection, we only take the abstract value.

**Figure 12:**
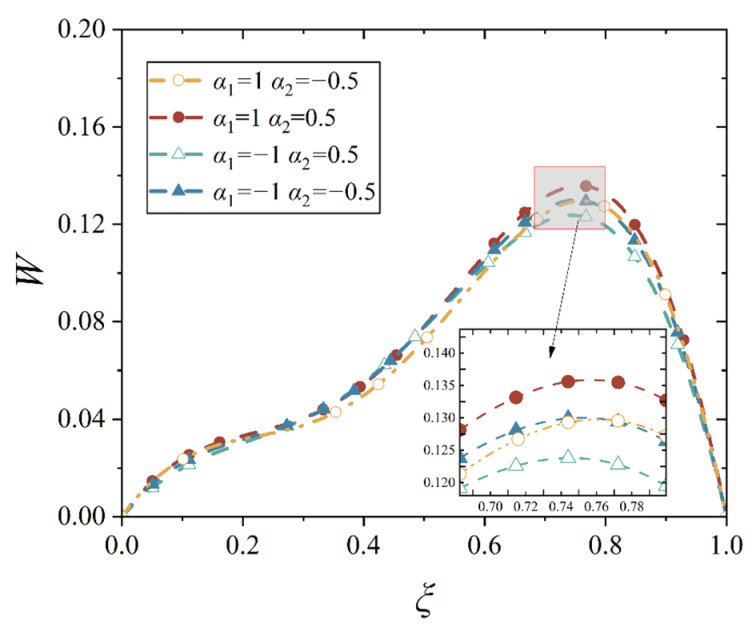
Deflection curves of simply supported conical shells with different gradient indices under mechanical load (*α* = 45°).

**Figure 13:**
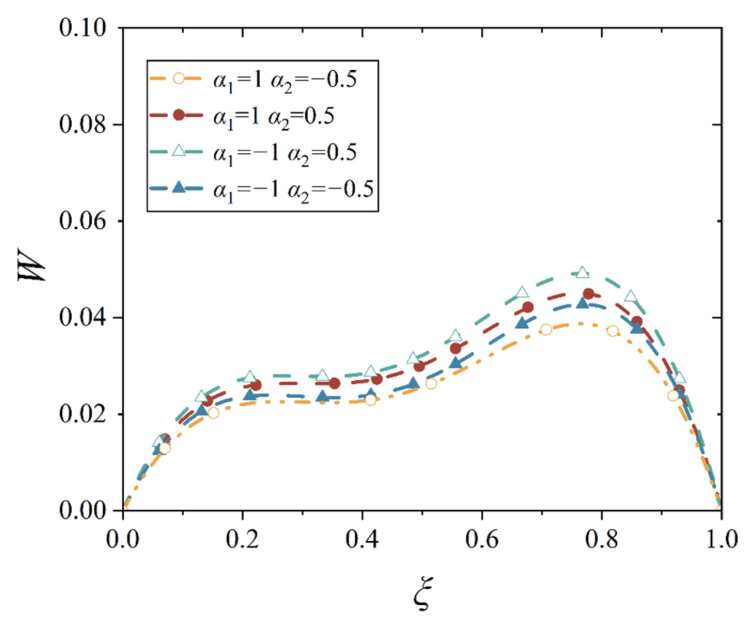
Deflection curves of simply supported conical shells with different gradient indices under thermal load (*α* = 45°).

**Figure 14:**
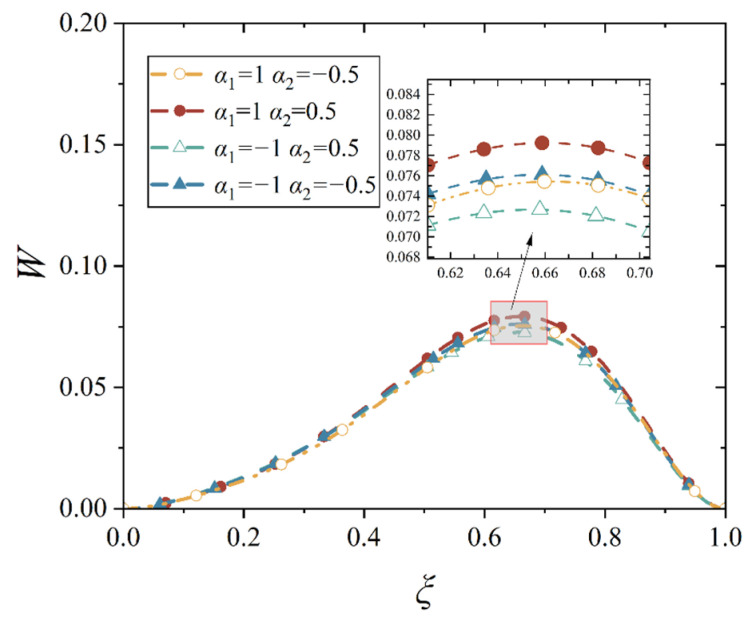
Deflection curves of fixed conical shells with different gradient indices under mechanical load (*α* = 45°).

**Figure 15:**
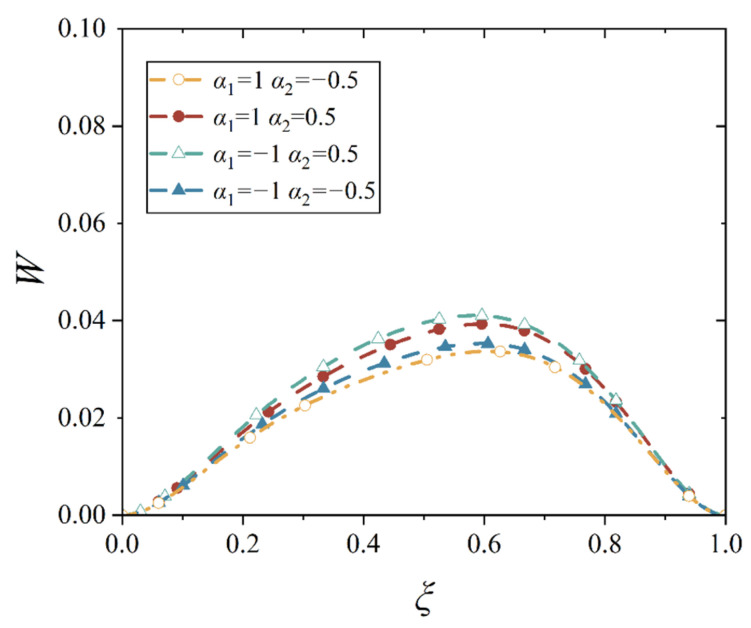
Deflection curves of fixed conical shells with different gradient indices under thermal load (*α* = 45°).

In our study, four different gradient indices are given, and they are *α*_1_ > *α*_2_ > 0, *α*_1_ < *α*_2_ < 0, *α*_1_ > 0 > *α*_2_, and *α*_2_ > 0 > *α*_1_, which stands for the four bimodular functionally graded cases, as shown in Table 1. With the four material cases, we plot the deflection curves of conical shell with two different boundary constraints and under mechanical load or thermal load, as shown in Figure 12, Figure 13, Figure 14 and Figure 15, in which, the half of cone apex angle is taken as 45°.

Although the deformation shapes of conical shells with two kinds of boundary constraints are different, the influence trends in different gradient indices are the same; this phenomenon may be easily observed from Figure 12, Figure 13, Figure 14 and Figure 15. The influence of different gradient indices on deformation under thermal load is slightly bigger than that under mechanical load, but their influence rules under the two types of loads are different. Under the mechanical load, the maximum displacement of the conical shell is the biggest in case *α*_1_ > *α*_2_ > 0, compared with other three cases, while the maximum displacement of the conical shell is the smallest in case *α*_2_ > 0 > *α*_1_. When the gradient index belongs to other two cases, that is, *α*_1_ > 0 > *α*_2_ and *α*_1_ < *α*_2_ < 0, the maximum displacement of the conical shell is close, but the displacement is slightly larger when *α*_1_ < *α*_2_ < 0. However, under the thermal load, this variation rule changes. When the gradient index case is *α*_2_ > 0 > *α*_1_, the maximum displacement of the conical shell becomes the biggest among the four cases, followed by *α*_1_ > *α*_2_ > 0, *α*_1_ < *α*_2_ < 0, and finally, *α*_1_ > 0 > *α*_2_, this physical phenomenon agrees with the basic rule that the greater the stiffness of the structure, the smaller the corresponding deformation under the same load. In addition, it can be observed from Figure 12, Figure 13, Figure 14 and Figure 15 that the position of the maximum displacement of the conical shell does not change significantly in different gradient cases.

**Figure 16:**
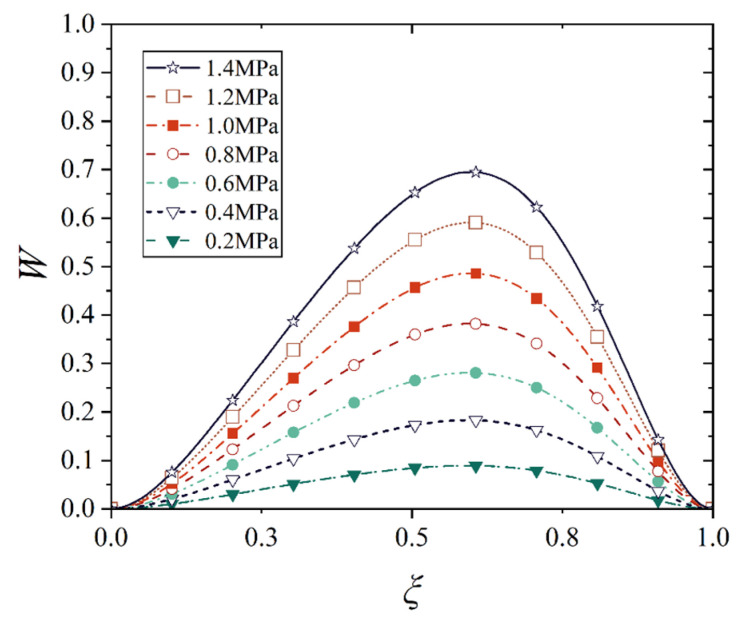
Deflection curves of fixed conical shell generatrix under increasing mechanical load (*α* = 70°).

**Figure 17:**
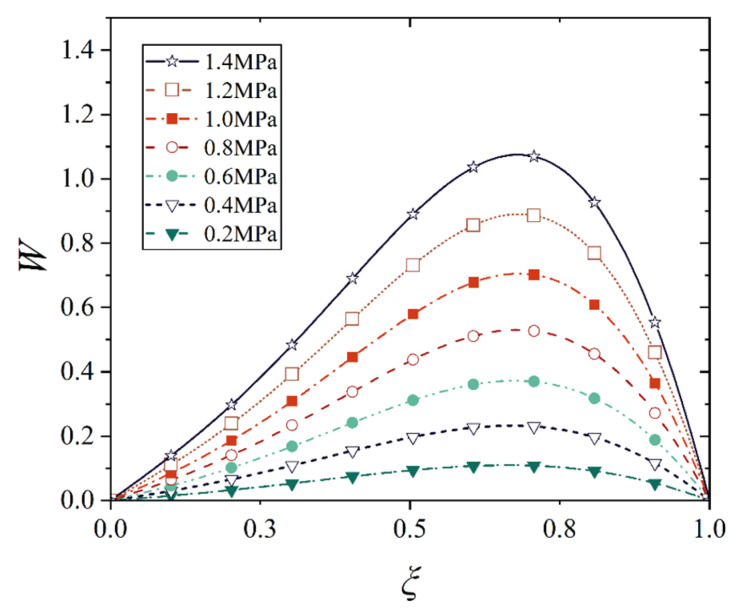
Deflection curves of simply supported conical shell generatrix under increasing mechanical load (*α* = 70°).

**Figure 18:**
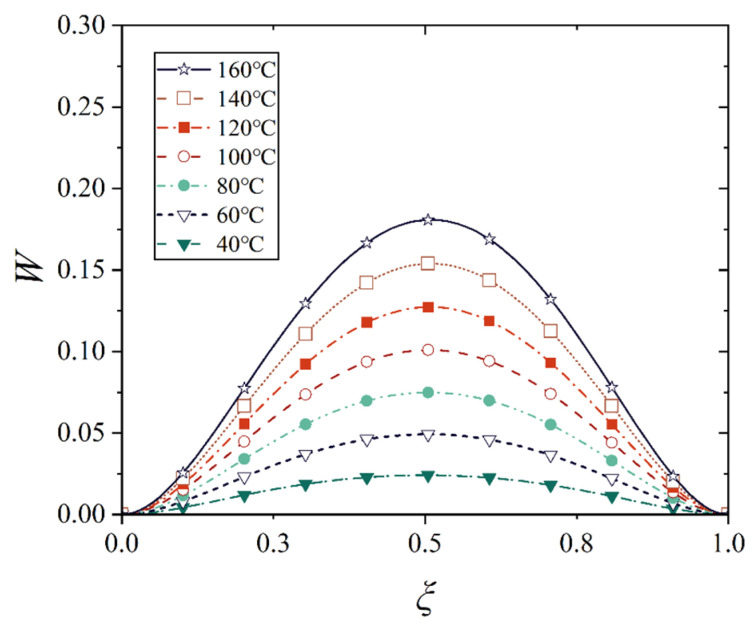
Deflection curves of fixed conical shell generatrix under increasing thermal load (*α* = 70°).

**Figure 19:**
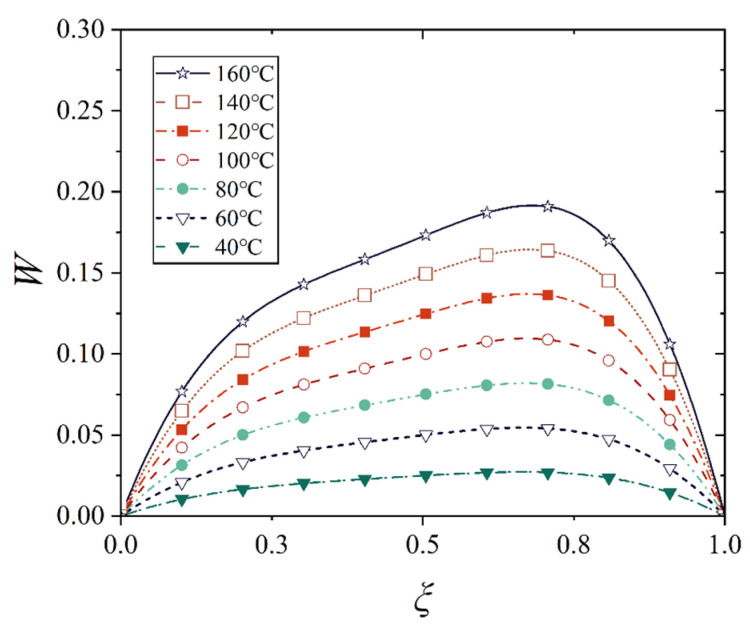
Deflection curves of simply supported conical shell generatrix under increasing thermal load (*α* = 70°).

Figure 16, Figure 17, Figure 18 and Figure 19 show the deflection curves of the generatrix of the conical shell with two ends fixed or simply supported, and under the increasing mechanical load or the increasing thermal load, in which the half of the cone apex angle is taken as 70°. It can be seen from Figure 16, Figure 17, Figure 18 and Figure 19 that with the increase in load, the deflection of the generatrix of the conical shell also increases, and the deflection variation with the increase in load is almost linear. However, it is interesting that the maximum displacement point of the simply supported conical shell is closer to the big end (*ξ* = 1) of the conical shell, while the maximum displacement point of the fixed conical shell is closer to the middle (*ξ* = 0.5) of the conical shell, and this law does not change significantly with the increase in load.

### 6.2. The Influence of Geometric Parameters on the W_max_ Position

In the truncated conical shell, there are several important geometric parameters that determine the shape of the truncated cone shell, such as the cone apex angle, the truncated distance, and the distance from the big end (cone bottom) to the small end (cone top). These geometric parameters have great influences on the maximum displacement and its position of the truncated conical shell.

**Figure 20:**
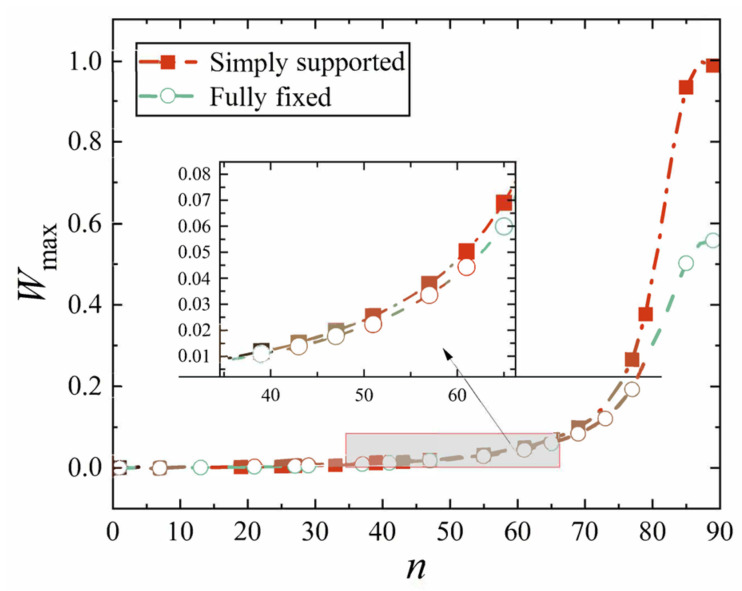
Variation of maximum displacement under mechanical load with cone apex angle.

**Figure 21:**
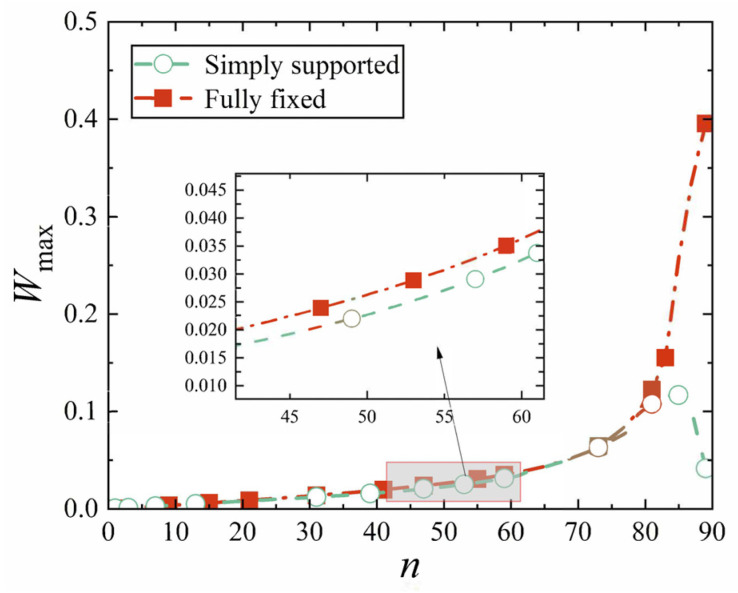
Variation of maximum displacement under thermal load with cone apex angle.

**Figure 22:**
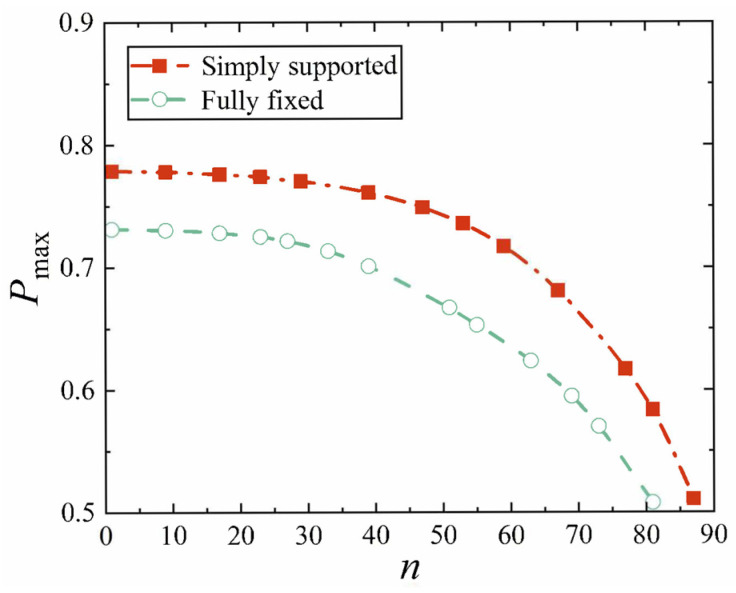
Variation of maximum displacement position under mechanical load with cone apex angle.

**Figure 23:**
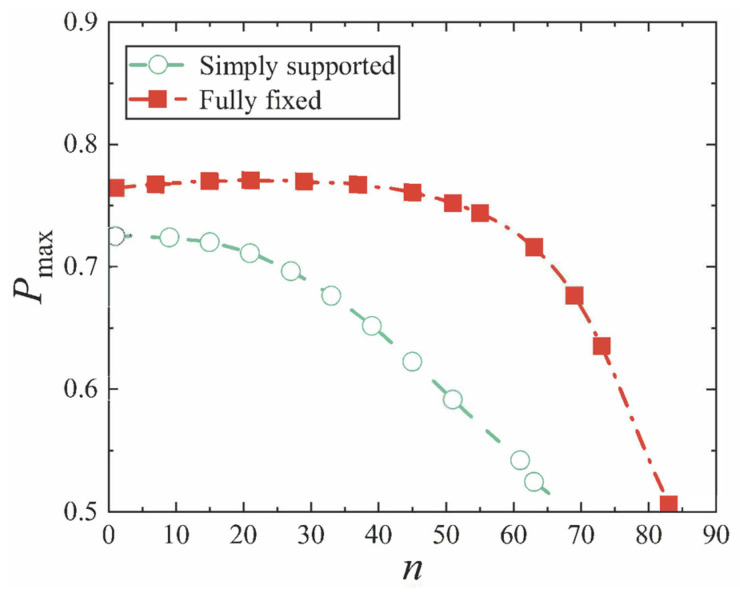
Variation of maximum displacement position under thermal load with cone apex angle.

First, we analyze the variation resulting from different cone apex angles. All other parameters are still provided in Table 2 and Table 3. The maximum displacement is still represented by *W*_max_, and the position of the maximum displacement of the conical shell is now represented by *P*_max_, and the cone top angle is represented by *n*. Figure 20, Figure 21, Figure 22 and Figure 23 show the variation of the maximum displacement and its position with the increase in cone top angle, still under two kinds of boundary constraints and two types of loads.

It is easily observed from Figure 20, Figure 21, Figure 22 and Figure 23 that, under mechanical load or thermal load, when the cone apex angle is less than 60°, the maximum displacement of the conical shell shows no significant change with respect to the angle. However, when the angle exceeds 60°, there is a rapid increase in the maximum displacement *W*_max_ of the conical shell. Furthermore, for the apex angles less than 60°, the maximum displacement of the simply supported conical shell consistently exceeds that of the fixed conical shell. The trend in the maximum displacements for conical shells with two ends fixed or simply supported remains largely similar; however, as the angle increases sufficiently, a clear divergence emerges between their respective maximum displacements. Ultimately, as this occurs, it becomes apparent that the maximum displacement of the simply supported conical shell gradually surpasses that of its fixed boundary counterpart. The reason for this phenomenon may be that the projection of the circumferential stress in the normal direction of the shell gradually decreases with the increase in the angle.

It is easy to see from Figure 22 and Figure 23 that with the increase in the cone apex angle, the maximum displacement point of the simply supported conical shell and the fixed conical shell gradually moves to the middle of the shell (*P*_max_ = 0.5), and the change is not obvious when the angle is small, but when the angle becomes bigger, the maximum displacement point moves rapidly. The change in the maximum displacement position of the conical shell with these two constraints is basically the same, but the difference exists in which the maximum displacement point of the fixed conical shell is always close to the middle of the conical shell.

**Figure 24:**
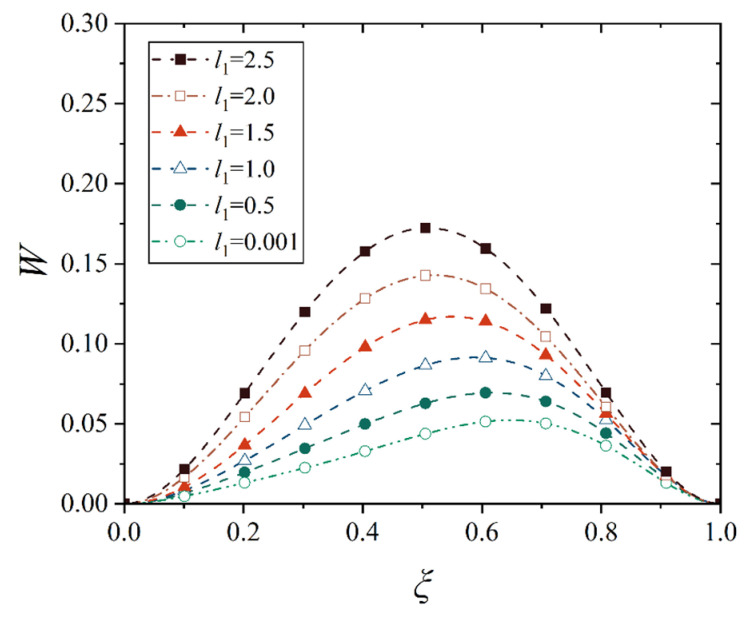
Deflection curves of fixed conical shells with different truncated distances *l*_1_ under mechanical load (*α* = 70°).

**Figure 25:**
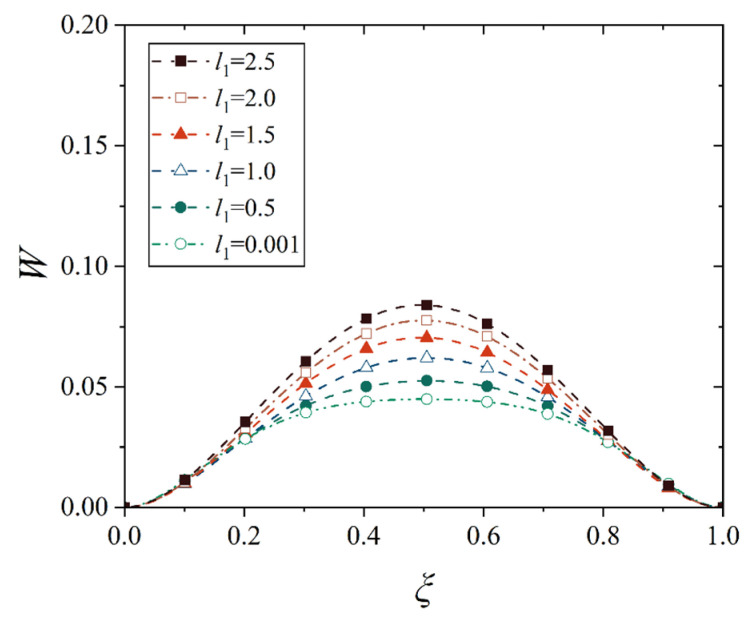
Deflection curves of fixed conical shells with different truncated distances *l*_1_ under thermal load (*α* = 70°).

**Figure 26:**
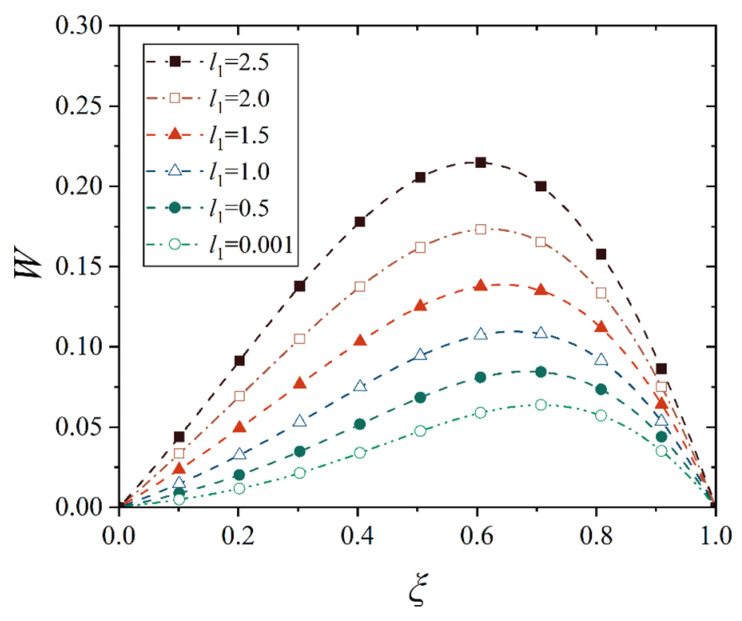
Deflection curves of simply supported conical shells with different truncated distances *l*_1_ under mechanical load (*α* = 70°).

**Figure 27:**
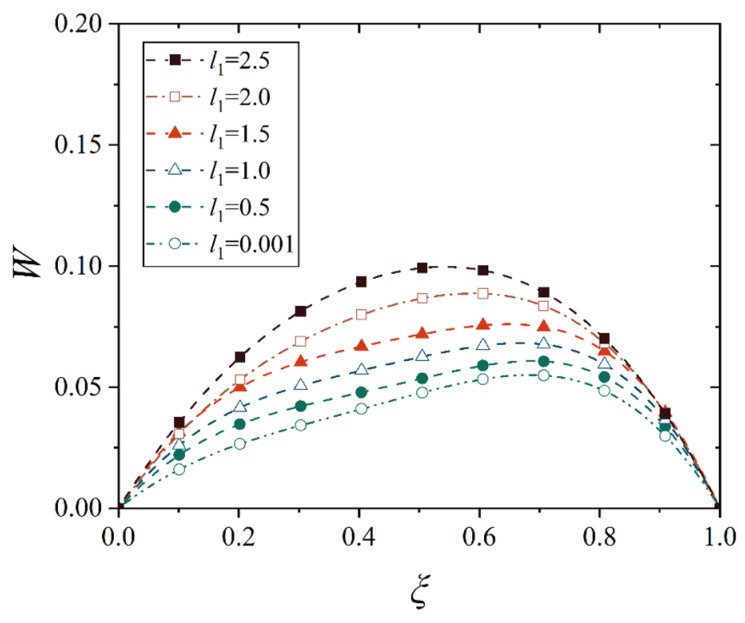
Deflection curves of simply supported conical shells with different truncated distances *l*_1_ under thermal load (*α* = 70°).

Next, we analyze the effect of truncated distance *l*_1_ (between the small end and the cone top) on the maximum displacement and its location of the conical shell. For this purpose, several representative values are selected to observe the change in the displacement of the conical shell, that is, *l*_1_ = 0, 0.5, 1.0, 1.5, 2.0, and 2.5. Note that the point at *l*_1_ = 0 is not in the domain of definition during the establishment of the geometric equation of the conical shell, so *l*_1_ = 0.001 is used here to stand for *l*_1_ = 0. In order to avoid the interference of the change in other geometric parameters, the length of the shell, that is, *l*_2_ − *l*_1_, is kept uniform for the conical shell with different truncated distances *l*_1_. Therefore, the deflection curves of the conical shell generatrix under two different constraints and two types of loads are shown in Figure 24, Figure 25, Figure 26 and Figure 27, in which the half of cone ape angle is taken as 70°.

It is readily observed from Figure 24, Figure 25, Figure 26 and Figure 27 that the maximum displacement of the conical shell under two boundary constraints gradually increases when the truncated distance *l*_1_ gradually increases, and this increase is approximately linear, and the position of the maximum displacement is gradually closer to the middle of the shell.

**Figure 28:**
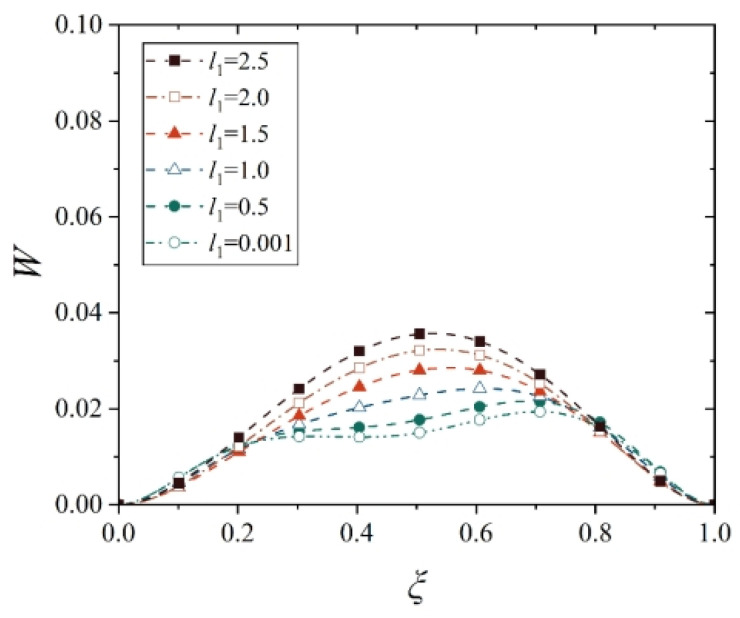
Deflection curves of fixed conical shells with different truncated distances *l*_1_ under thermal load (*α* = 45°).

With the small end gradually away from the top of the cone, the shape of the conical shell deformation has undergone a huge change, the displacement of the middle of the shell gradually becomes prominent, the displacement change on both sides of the middle is also gradually approaching, forming a deformation similar to the plate structure, that is, when the truncated distance *l*_1_ is large enough, the deformation characteristics of the conical shell will be lost, namely, the deformation is smaller near the small end and the deformation is larger near the big end, and the maximum deformation is close to the big end. In Figure 25, it is found that in conical shells with different truncated distances *l*_1_, the change in the maximum displacement position is not obvious under thermal load, which is due to the fact that the maximum displacement position of the fixed conical shell has reached the middle of the shell and no longer moved significantly when the cone apex angle is 70°. As a comparison, Figure 28 shows the change in deflection curves with the truncated distance *l*_1_ at a cone apex angle of 45° under thermal load. It is easily observed that the movement rule of its maximum displacement position is similar to Figure 25.

### 6.3. The Combined Influence of Mechanical and Thermal Loads

**Figure 29:**
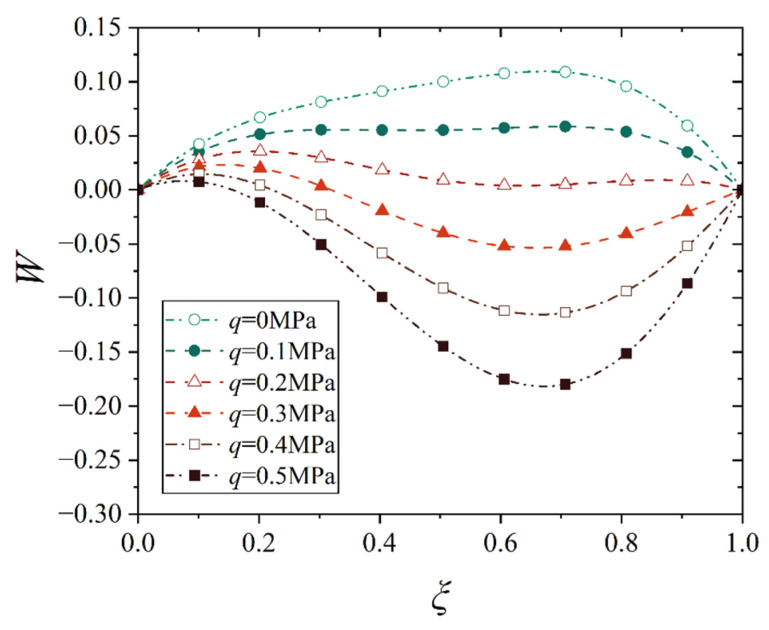
Deflection curves of simply supported conical shells with gradual increase in mechanical load at an external temperature *T*_1_ of 100 °C (*α* = 70°).

**Figure 30:**
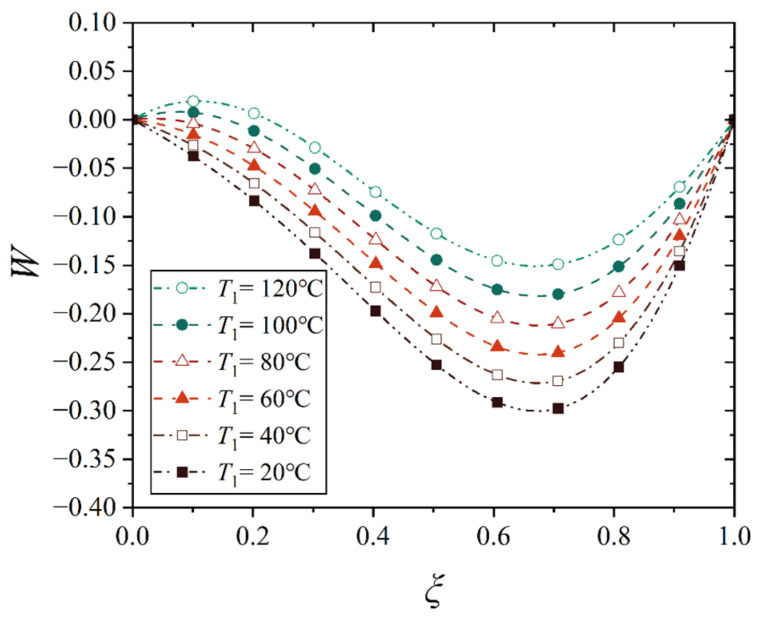
Deflection curves of simply supported conical shells with gradual increase in external temperature when the mechanical load *q* is 0.5 MPa (*α* = 70°).

In the above discussion, for the convenience of analysis, we do not consider the direction of structural deformation caused by the mechanical load or thermal load, that is, we only use the absolute value to express the magnitude of deformation, not considering positive values or negative values. This is feasible only when a single load acts. When the mechanical and thermal loads act together, the superposition of deformation is inevitably involved, so the absolute value of deformation cannot be used anymore. In this case, the positive or negative values of deflection should be considered. For this purpose, we still follow the basic parameters in Table 2 and Table 3, and compute and plot the deflection curves of the simply supported conical shell under the combined action of mechanical load and thermal load, as shown in Figure 29 and Figure 30, in which Figure 29 shows the deflection curves under the action of different mechanical loads when the external temperature is 100 °C, while Figure 30 shows the deflection curves under different external temperatures when the mechanical load is 0.5 MPa.

From Figure 29 and Figure 30, it is easy to see that the deflection of the shell is always positive when the external temperature rises, and the deflection value of the shell is always negative when the external pressure is applied. Therefore, when the two types of loads are superimposed, the influences of two types of loads will partially offset. When the conical shell is under the combined action of mechanical load and thermal load, there will be both positive and negative deflection values on the shell, which depends on their relative magnitude. For example, the deflection values in Figure 29 present as positive and negative, while the deflection values in Figure 30 are totally negative, except for the influence under a higher external temperature, for example, the curve of *T*_1_ = 120 °C has positive deflection values in a local range.

## 7. Conclusions

In this study, we theoretically analyze the large deflection problem of bimodular functionally graded truncated thin conical shells under the combined action of transverse mechanical load and non-uniform thermal load, and obtain the variation solution under two different boundary constraints. The numerical simulation based on Abaqus also validates the variation solution obtained. The influences of important parameters on the deformation are discussed in detail; these parameters include bimodular functionally graded indices that take into account material aspects, the cone apex angle and the truncated distance of conical shells that consider the geometry aspect, as well as the load aspect. The following conclusions can be drawn:

1. The introduction of bimodular functionally graded material will affect the maximum displacement of the conical shell to a certain extent, and this effect has different rules for conical shell under mechanical load and thermal load, but keeps the same rule under different boundary constraints.
2. The maximum displacement of the conical shell is closely related to the cone apex angle. For the smaller cone apex angle, the displacement is dominated by compressive stress, thus no large displacement occurs. However, high values of the cone apex angle generate more bending stress, and this generates different deflections, thus influencing the maximum displacement and its position. At the same time, the maximum displacement of simply supported conical shell is slightly larger than that of the fixed conical shell.
3. With the increase in the truncated distance *l*_1_, the maximum displacement of the conical shell under two kinds of boundary constraints and two types of loads will gradually increase, and its position will also move closer to the middle of the shell. When the distance *l*_1_ is large enough, the conical shell will lose the deformation characteristics it should have, that is, the deflection at one end is smaller while the deflection at another end is larger, and the maximum deflection is close to the large end. Finally, the conical shell will have the deformation characteristics of plates or cylindrical shells.
4. An external temperature rise causes the positive displacement of the conical shell, while external mechanical pressure causes the negative displacement. When the mechanical load (external pressure) and the thermal load (temperature rise) act together, part of the displacement will be offset, so that the conical shell may have positive or negative displacement at the same time.

This work is helpful to analyze and design functionally graded truncated thin conical shells under a thermal environment when exploring the mechanical potential of bimodular materials has become a development trend. Although the theoretical solution presented in this study is validated by the numerical simulation, its experimental verification is still necessary. Meanwhile, the theoretical solution is based on the known bimodular parameters, so further testing for bimodular parameters of materials is also necessary. Finally, we would also like to point out that there are also uncertainties in the input parameters, and thus, it is interesting to carry out uncertainty analysis quantifying the influence of all uncertain input parameters with respect to all uncertain output parameters. In this case, DNN (deep neural networks), a complex model based on multilayer perceptron, is a good choice and future research direction in this field.

## Figures and Tables

**Table 1:** Four bimodular cases and corresponding functional gradient indices.

Bimodular Case Gradient Index	*E^+^* > *E*_0_ > *E*^−^ *α*_1_ > *α*_2_ > 0	*E^+^* < *E*_0_ < *E*^−^ *α*_1_ < *α*_2_ < 0	*E^+^* > *E*_0_, *E^−^* > *E*_0_ *α*_1_ > 0 > *α*_2_	*E^+^* < *E*_0_, *E^−^* < *E*_0_ *α*_1_ < 0 < *α*_2_
*α*_1_	1	−1	1	−1
*α*_2_	0.5	−0.5	−0.5	0.5

**Table 2:** Bimodular functionally graded material parameters and heat-related parameters.

*E*_0_ (MPa)	*μ^+^*	*μ* ^−^	*α*_0_ (/°C)	*K*_0_ (W/mk)	*α*_3_
2 × 10^4^	0.4	0.2	1.5 × 10^5^	100	0.75

**Table 3:** Geometric parameters and load parameters of truncated conical shells.

*l*_1_ (m)	*l*_2_ (m)	*δ* (m)	*α*	*q* (MPa)	*T*_0_ (°C)	*T*_1_ (°C)	*T*_2_ (°C)
1	5	0.1	7π/18	1	20	20	70

**Table 4:** Tensile and compressive thicknesses under different gradient indices.

Thickness	*α*_1_ = 1, *α*_2_ = 0.5	*α*_1_ = −1, *α*_2_ = −0.5	*α*_1_ = 1, *α*_2_ = −0.5	*α*_1_ = −1, *α*_2_ = 0.5
*δ*_1_ (m)	0.05833952172	0.04581547570	0.05446972327	0.04980533659
*δ*_2_ (m)	0.04166047828	0.05418452430	0.04553027673	0.05019466341

## Data Availability

Data are contained within the article.

## Appendix A

$$\begin{array}{cc} f_{1} & =\pi D_{1}^{-}\sin\alpha \left[2\ln(l_{2}){l_{1}}^{3}{l_{2}}^{2}-2\ln(l_{2}){l_{1}}^{3}{l_{2}}^{2}+2{l_{1}}^{2}{l_{2}}^{3}+\frac{1}{10}{l_{1}}^{5}+\frac{1}{15}{l_{1}}^{5}-\frac{2}{3}{l_{1}}^{3}{l_{2}}^{2}-{l_{1}}^{4}l_{2}-\frac{1}{2}l_{1}{l_{2}}^{4}\right]\\ & +\pi D_{1}^{+}\sin\alpha \left(\frac{7}{30}{l_{2}}^{5}+\frac{1}{10}{l_{1}}^{5}+l_{1}^{2}l_{2}^{3}-\frac{1}{3}{l_{1}}^{3}{l_{2}}^{2}-\frac{5}{6}l_{1}{l_{2}}^{4}-\frac{1}{6}{l_{1}}^{4}l_{2}\right) \end{array}$$

$$\begin{array}{cc} f_{2} & =\pi D_{1}^{+}\sin\alpha [(\frac{1}{35}-\frac{1}{70}\mu^{+})({l_{1}}^{8}l_{2}-l_{1}{l_{2}}^{8})+(\frac{4}{35}-\frac{2}{35}\mu^{+})({l_{1}}^{2}{l_{2}}^{7}-{l_{1}}^{7}{l_{2}}^{2})\\ & +(\frac{2}{5}-\frac{1}{5}\mu^{+})({l_{1}}^{4}{l_{2}}^{5}-{l_{1}}^{5}{l_{2}}^{4})+(\frac{4}{15}-\frac{2}{15}\mu^{+})({l_{1}}^{6}{l_{2}}^{3}-{l_{1}}^{3}{l_{2}}^{6})+(\frac{1}{315}-\frac{1}{630}\mu^{+})({l_{2}}^{9}-{l_{1}}^{9})]\\ & +\pi D_{1}^{-}\mu^{-}\sin\alpha [\frac{1}{5}({l_{1}}^{5}{l_{2}}^{4}-{l_{1}}^{4}{l_{2}}^{5})+\frac{2}{15}({l_{1}}^{3}{l_{2}}^{6}-{l_{1}}^{6}{l_{2}}^{3})+\frac{2}{35}({l_{1}}^{7}{l_{2}}^{2}-{l_{1}}^{2}{l_{2}}^{7})\\ & +\frac{1}{70}(l_{1}{l_{2}}^{8}-{l_{1}}^{8}l_{2})+\frac{1}{630}({l_{1}}^{9}-{l_{2}}^{9})] \end{array}$$

$$\begin{array}{cc} f_{3} & =\pi D_{1}^{-}\sin\alpha \left[\frac{1}{12}\left({l_{2}}^{4}-{l_{1}}^{4}\right)-\ln(l_{1}){l_{1}}^{2}{l_{2}}^{2}+\ln(l_{2}){l_{1}}^{2}{l_{2}}^{2}+\frac{2}{3}\left({l_{1}}^{3}l_{2}-l_{1}{l_{2}}^{3}\right)\right]\\ & +\pi D_{1}^{+}\sin\alpha \left(\frac{1}{6}{l_{2}}^{4}-\frac{1}{6}{l_{1}}^{4}-\frac{1}{3}l_{1}{l_{2}}^{3}+\frac{1}{3}{l_{1}}^{3}l_{2}\right) \end{array}$$

$$\begin{array}{cc} f_{4} & =\pi D_{1}^{+}\mu^{+}\cos\alpha [\frac{1}{210}({l_{1}}^{7}-{l_{2}}^{7})+\frac{1}{6}({l_{1}}^{3}{l_{2}}^{4}-{l_{2}}^{3}{l_{1}}^{4})+\frac{1}{30}(l_{1}{l_{2}}^{6}-l_{2}{l_{1}}^{6})+\frac{1}{10}({l_{2}}^{2}{l_{1}}^{5}-{l_{1}}^{2}{l_{2}}^{5})]\\ & +\pi D_{1}^{-}\cos\alpha [(2+\frac{\mu^{-}}{6}){l_{1}}^{3}{l_{2}}^{4}-(\frac{1}{2}+\frac{\mu^{-}}{6}){l_{2}}^{3}{l_{1}}^{4}+(\frac{\mu^{-}}{10}-\frac{6}{5}){l_{2}}^{2}{l_{1}}^{5}-(\frac{3}{5}+\frac{\mu^{-}}{10}){l_{1}}^{2}{l_{2}}^{5}\\ & +(\frac{1}{5}-\frac{\mu^{-}}{30})l_{2}{l_{1}}^{6}+(\frac{2}{15}+\frac{\mu^{-}}{30})l_{1}{l_{2}}^{6}+(\frac{\mu^{-}}{210}-\frac{2}{105}){l_{1}}^{7}-(\frac{1}{70}+\frac{\mu^{-}}{210}){l_{2}}^{7}+(2\ln(l_{1})-2\ln(l_{2})){l_{2}}^{3}{l_{1}}^{4}] \end{array}$$

$$\begin{array}{cc} f_{5} & =\pi D_{1}^{+}\sin\alpha [(\frac{2}{21}\mu^{+}-\frac{4}{21})({l_{1}}^{3}{l_{2}}^{7}+{l_{1}}^{7}{l_{2}}^{3})+(\frac{1}{14}-\frac{1}{28}\mu^{+})({l_{1}}^{2}{l_{2}}^{8}+{l_{1}}^{8}{l_{2}}^{2})+(\frac{1}{5}\mu^{+}-\frac{2}{5}){l_{1}}^{5}{l_{2}}^{5}\\ & +(\frac{1}{126}\mu^{+}-\frac{1}{63})(l_{1}{l_{2}}^{9}+{l_{1}}^{9}l_{2})+(\frac{1}{3}-\frac{1}{6}\mu^{+})({l_{1}}^{4}{l_{2}}^{6}+{l_{1}}^{6}{l_{2}}^{4})+(\frac{1}{630}-\frac{1}{1260}\mu^{+})({l_{1}}^{10}+{l_{2}}^{10})]\\ & +\pi D_{1}^{-}\mu^{-}\sin\alpha [\frac{1}{126}({l_{1}}^{9}l_{2}+{l_{2}}^{9}l_{1})-\frac{1}{6}({l_{1}}^{6}{l_{2}}^{4}+{l_{1}}^{4}{l_{2}}^{6})+\frac{2}{21}({l_{1}}^{7}{l_{2}}^{3}+{l_{1}}^{3}{l_{2}}^{7})-\frac{1}{28}({l_{1}}^{8}{l_{2}}^{2}+{l_{1}}^{2}{l_{2}}^{8})\\ & -\frac{1}{1260}({l_{1}}^{10}+{l_{2}}^{10})+\frac{1}{5}{l_{1}}^{5}{l_{2}}^{5}] \end{array}$$

$$\begin{array}{cc} f_{6} & =\pi D_{1}^{+}\sin\alpha (-\frac{7}{15}l_{1}{l_{2}}^{5}+\frac{1}{15}{l_{1}}^{5}l_{2}-\frac{1}{30}{l_{1}}^{6}+\frac{1}{10}{l_{2}}^{6}+\frac{5}{6}{l_{1}}^{2}{l_{2}}^{4}+\frac{1}{6}{l_{1}}^{4}{l_{2}}^{2}-\frac{2}{3}{l_{1}}^{3}{l_{2}}^{3})\\ & +\pi D_{1}^{-}\sin\alpha [\frac{1}{60}{l_{2}}^{6}-\frac{1}{30}{l_{1}}^{6}+\frac{7}{12}{l_{1}}^{4}{l_{2}}^{2}-\frac{4}{3}{l_{1}}^{3}{l_{2}}^{3}+\frac{1}{2}{l_{1}}^{2}{l_{2}}^{4}-\ln(l_{1}){l_{1}}^{4}{l_{2}}^{2}+\ln(l_{2}){l_{1}}^{4}{l_{2}}^{2}-\frac{2}{15}l_{1}{l_{2}}^{5}\\ & +\frac{2}{5}{l_{1}}^{5}l_{2}]+\pi D_{1}^{+}\sin\alpha (\frac{1}{6}{l_{2}}^{4}-\frac{1}{6}{l_{1}}^{4}-\frac{1}{3}l_{1}{l_{2}}^{3}+\frac{1}{3}{l_{1}}^{3}l_{2}) \end{array}$$

$$\begin{array}{cc} f_{7} & =\pi D_{1}^{+}\mu^{+}\cos\alpha \left[\frac{2}{15}\left({l_{1}}^{6}{l_{2}}^{5}-{l_{1}}^{5}{l_{2}}^{6}\right)+\frac{1}{315}\left({l_{1}}^{10}l_{2}-l_{1}{l_{2}}^{10}\right)+\frac{1}{63}\left({l_{1}}^{2}{l_{2}}^{9}-{l_{1}}^{9}{l_{2}}^{2}\right)+\frac{1}{21}\left({l_{1}}^{8}{l_{2}}^{3}-{l_{1}}^{3}{l_{2}}^{8}\right)\right.\\ & \left.+\frac{2}{21}\left({l_{1}}^{4}{l_{2}}^{7}-{l_{1}}^{7}{l_{2}}^{4}\right)+\frac{1}{3465}\left({l_{2}}^{11}-{l_{1}}^{11}\right)\right] \end{array}$$

$$\begin{array}{cc} f_{8} & =\pi D_{1}^{+}\sin\alpha [\frac{1}{15015}({l_{2}}^{14}-{l_{1}}^{4})+\frac{4}{5005}({l_{1}}^{13}l_{2}-l_{1}{l_{2}}^{13})+\frac{1}{35}({l_{1}}^{4}{l_{2}}^{10}-{l_{2}}^{4}{l_{1}}^{10})+\frac{1}{35}({l_{1}}^{6}{l_{2}}^{8}-{l_{1}}^{8}{l_{2}}^{6})\\ & +\frac{4}{105}({l_{1}}^{9}{l_{2}}^{5}-{l_{1}}^{5}{l_{2}}^{9})+\frac{16}{1155}({l_{2}}^{3}{l_{1}}^{11}-{l_{1}}^{3}{l_{2}}^{11})+\frac{1}{231}({l_{1}}^{2}{l_{2}}^{12}-{l_{1}}^{12}{l_{2}}^{2})]\\ & +\pi D_{1}^{-}\mu^{-}\cos\alpha [\frac{1}{315}({l_{1}}^{10}l_{2}-{l_{2}}^{10}l_{1})+\frac{1}{63}({l_{2}}^{9}{l_{1}}^{2}-{l_{1}}^{9}{l_{2}}^{2})+\frac{2}{15}({l_{1}}^{6}{l_{2}}^{5}-{l_{1}}^{5}{l_{2}}^{6})+\frac{2}{21}({l_{1}}^{4}{l_{2}}^{7}-{l_{1}}^{7}{l_{2}}^{4})\\ & +\frac{1}{21}({l_{1}}^{8}{l_{2}}^{3}-{l_{1}}^{3}{l_{2}}^{8})+\frac{1}{3465}({l_{2}}^{11}-{l_{1}}^{11}) \end{array}$$

$$f_{9}=\pi D_{1}^{-}\cos\alpha \left[2\ln(l_{2}){l_{1}}^{3}{l_{2}}^{3}-2\ln(l_{1}){l_{1}}^{3}{l_{2}}^{3}+\frac{1}{30}\left({l_{1}}^{6}-{l_{2}}^{6}\right)+\frac{3}{10}\left(l_{1}{l_{2}}^{5}-{l_{1}}^{5}l_{2}\right)+\frac{3}{2}\left({l_{1}}^{4}{l_{2}}^{2}-{l_{1}}^{2}{l_{2}}^{4}\right)\right]$$

$$\begin{array}{cc} f_{10} & =\pi D_{1}^{-}\cos\alpha \cot\alpha [\ln(l_{1}){l_{1}}^{4}{l_{2}}^{4}-\ln(l_{1}){l_{1}}^{4}{l_{2}}^{4}+\frac{1}{280}({l_{2}}^{8}-{l_{1}}^{8})+\frac{4}{5}({l_{1}}^{5}{l_{2}}^{3}-{l_{1}}^{3}{l_{2}}^{5})+\frac{1}{5}({l_{1}}^{2}{l_{2}}^{6}-{l_{1}}^{6}{l_{2}}^{2})\\ & +\frac{4}{105}(l_{2}{l_{1}}^{7}-{l_{2}}^{7}l_{1})]+\pi \sin\alpha (D_{2}^{+}+D_{2}^{-})\{\frac{13}{3}({l_{1}}^{4}{l_{2}}^{2}-{l_{1}}^{2}{l_{2}}^{4})+\frac{7}{15}({l_{2}}^{6}-{l_{1}}^{6})+\frac{38}{15}({l_{1}}^{5}l_{2}-l_{1}{l_{2}}^{5})\\ & +(4{l_{1}}^{4}{l_{2}}^{2}+8{l_{1}}^{3}{l_{2}}^{3}+4{l_{1}}^{2}{l_{2}}^{4})[\ln(l_{2})-\ln(l_{1})]\} \end{array}$$

$$\begin{array}{cc} g_{1} & =2\pi M_{T}\cot\alpha \left.\left[\frac{1}{3}\left({l_{2}}^{3}-{l_{1}}^{3}\right)-2l_{1}{l_{2}}^{2}\ln(l_{2})+2{l_{1}}^{2}l_{2}\ln(l_{1})+2l_{1}{l_{2}}^{2}\ln(l_{1})-2{l_{1}}^{2}l_{2}\ln(l_{2})\right.+3\left(l_{1}{l_{2}}^{2}-{l_{1}}^{2}l_{2}\right)\right]\\ & +2\pi N_{T}\cos\alpha \left[\frac{1}{3}\left({l_{1}}^{2}{l_{2}}^{3}-1{l_{1}}^{3}{l_{2}}^{2}\right)+\frac{1}{6}\left({l_{1}}^{4}l_{2}-l_{1}{l_{2}}^{4}\right)+\frac{1}{30}\left({l_{2}}^{5}-{l_{1}}^{5}\right)\right] \end{array}$$

$$g_{2}=2\pi N_{T}\cot\alpha \left[\frac{1}{210}\left({l_{2}}^{8}-{l_{1}}^{8}\right)+\frac{1}{35}\left({l_{1}}^{7}l_{2}-l_{1}{l_{2}}^{7}\right)+\frac{1}{15}\left({l_{1}}^{2}{l_{2}}^{6}-{l_{1}}^{6}{l_{2}}^{2}\right)+\frac{1}{15}\left({l_{1}}^{5}{l_{2}}^{3}-{l_{1}}^{3}{l_{2}}^{5}\right)\right]$$

$$\begin{array}{cc} q_{1} & =2\pi q\sin\alpha \left[\frac{1}{2}{l_{1}}^{2}{l_{2}}^{2}\left({l_{2}}^{2}-{l_{1}}^{2}\right)-\frac{2}{5}(l_{1}+l_{2})\left({l_{2}}^{5}-{l_{1}}^{5}\right)+\frac{1}{4}\left({l_{1}}^{2}+4l_{1}l_{2}+{l_{2}}^{2}\right)\left({l_{2}}^{4}-{l_{1}}^{4}\right)\right.\\ & \left.+\frac{1}{6}\left({l_{1}}^{6}+{l_{2}}^{6}\right)-\frac{2}{3}\left({l_{1}}^{2}l_{2}+l_{1}{l_{2}}^{2}\right)\left({l_{2}}^{3}-{l_{1}}^{3}\right)\right] \end{array}$$

$$\xi_{0}=-g_{1}-q_{1}$$

$$\begin{array}{cc} \xi_{1} & =\frac{-2}{{\left(f_{1}^{2}-4f_{3}f_{6}\right)}^{2}}\left({f_{1}}^{2}f_{3}f_{4}^{2}-f_{1}^{2}f_{6}f_{9}^{2}+4f_{3}f_{6}^{2}f_{9}^{2}+4f_{6}f_{3}^{2}f_{4}^{2}+f_{1}^{3}f_{4}f_{9}-4f_{1}f_{3}f_{4}f_{6}f_{9}\right)\\ & +\frac{4}{f_{1}^{2}-4f_{3}f_{6}}\left(f_{4}^{2}f_{3}+f_{9}^{2}f_{6}-f_{4}f_{1}f_{9}\right)+2f_{10}-2g_{2} \end{array}$$

$$\begin{array}{cc} \xi_{2} & =\frac{-3}{({f_{1}}^{2}-4f_{3}f_{6}{)}^{2}}(2{f_{1}}^{2}f_{2}f_{6}f_{9}-2{f_{1}}^{2}f_{3}f_{4}f_{5}+8f_{3}f_{2}{f_{6}}^{2}f_{9}+8f_{6}{f_{3}}^{2}f_{4}f_{5}+{f_{1}}^{3}f_{2}f_{4}+{f_{1}}^{3}f_{5}f_{9}\\ & -4f_{1}f_{2}f_{3}f_{4}f_{6}-4f_{1}f_{3}f_{5}f_{6}f_{9})+\frac{12}{{f_{1}}^{2}-4f_{3}f_{6}}(f_{3}f_{4}f_{5}-f_{1}f_{5}f_{9}-f_{4}f_{1}f_{2}+f_{9}f_{2}f_{6})+3f_{5}\\ & +\pi D_{1}^{+}\sin\alpha (\frac{1}{6}{l_{2}}^{4}-\frac{1}{6}{l_{1}}^{4}-\frac{1}{3}l_{1}{l_{2}}^{3}+\frac{1}{3}{l_{1}}^{3}l_{2}) \end{array}$$

$$\begin{array}{cc} \xi_{3} & =\frac{4}{{\left(f_{1}^{2}-4f_{3}f_{6}\right)}^{2}}\left(4f_{3}f_{2}^{2}f_{6}^{2}+4f_{6}f_{3}^{2}f_{5}^{2}+f_{1}^{3}f_{2}f_{5}-f_{1}^{2}f_{2}^{2}f_{6}-f_{1}^{2}f_{3}f_{5}^{2}-4f_{1}f_{2}f_{3}f_{5}f_{6}\right)\\ & +\frac{8}{f_{1}^{2}-4f_{3}f_{6}}\left(f_{2}^{2}f_{6}+f_{3}f_{5}^{2}-f_{2}f_{1}f_{5}\right)+4f_{7} \end{array}$$

## Appendix B

$$\begin{array}{cc} h_{1} & =\pi D_{1}^{+}\sin\alpha [(\frac{7\mu^{+}}{288}-\frac{83}{720})({l_{1}}^{5}{l_{2}}^{4}-{l_{1}}^{4}{l_{2}}^{5})+(\frac{7\mu^{+}}{432}-\frac{83}{1080})({l_{1}}^{3}{l_{2}}^{6}-{l_{1}}^{6}{l_{2}}^{3})\\ & +(\frac{\mu^{+}}{144}-\frac{83}{2520})({l_{1}}^{7}{l_{2}}^{2}-{l_{1}}^{2}{l_{2}}^{7})+(\frac{\mu^{+}}{576}-\frac{83}{10080})(l_{1}{l_{2}}^{8}-{l_{1}}^{8}l_{2})+(\frac{\mu^{+}}{5184}-\frac{83}{90720})({l_{1}}^{9}-{l_{2}}^{9})]\\ & +\pi D_{1}^{-}\mu^{-}\sin\alpha [\frac{1}{576}(l_{1}{l_{2}}^{8}-{l_{1}}^{8}l_{2})+\frac{7}{432}({l_{1}}^{3}{l_{2}}^{6}-{l_{1}}^{6}{l_{2}}^{3})+\frac{7}{288}({l_{1}}^{5}{l_{2}}^{4}-{l_{1}}^{4}{l_{2}}^{5})\\ & +\frac{1}{144}({l_{1}}^{7}{l_{2}}^{2}-{l_{1}}^{2}{l_{2}}^{7})+\frac{1}{5184}({l_{1}}^{9}-{l_{2}}^{9})]+\pi D_{1}^{+}\sin\alpha (\frac{1}{6}{l_{2}}^{4}-\frac{1}{6}{l_{1}}^{4}-\frac{1}{3}l_{1}{l_{2}}^{3}+\frac{1}{3}{l_{1}}^{3}l_{2}) \end{array}$$

$$\begin{array}{cc} h_{2} & =\pi D_{1}^{+}\sin\alpha \left(\frac{1}{6}{l_{1}}^{4}l_{2}-\frac{1}{3}{l_{2}}^{2}{l_{1}}^{3}+{l_{2}}^{3}{l_{1}}^{2}-\frac{5}{6}{l_{2}}^{4}l_{1}+\frac{1}{10}{l_{1}}^{5}+\frac{7}{30}{l_{2}}^{5}\right)\\ & +\pi D_{1}^{-}\sin\alpha \left[\frac{1}{15}{l_{2}}^{5}-{l_{1}}^{4}l_{2}-\frac{2}{3}\right.{l_{2}}^{2}{l_{1}}^{3}+2{l_{2}}^{3}{l_{1}}^{2}\left.-\frac{1}{2}{l_{2}}^{4}l_{1}+\frac{1}{10}{l_{1}}^{5}-2\ln(l_{2}){l_{2}}^{2}{l_{1}}^{3}+2\ln(l_{1}){l_{2}}^{3}{l_{1}}^{2}\right] \end{array}$$

$$\begin{array}{cc} h_{3} & =\pi D_{1}^{+}\sin\alpha \left[\frac{1}{3}\left({l_{1}}^{3}l_{2}-{l_{2}}^{3}l_{1}\right)+\frac{1}{6}\left({l_{2}}^{4}-{l_{1}}^{4}\right)\right]\\ & +\pi D_{1}^{-}\sin\alpha \left[\frac{1}{15}{l_{2}}^{5}+\frac{1}{10}{l_{1}}^{5}+2{l_{2}}^{3}{l_{1}}^{2}-2\ln(l_{2}){l_{1}}^{3}{l_{2}}^{2}\right.\left.+2\ln(l_{1}){l_{1}}^{3}{l_{2}}^{2}-\frac{2}{3}{l_{1}}^{3}{l_{2}}^{2}-\frac{1}{2}{l_{2}}^{4}l_{1}-{l_{1}}^{4}l_{2}\right] \end{array}$$

$$\begin{array}{cc} h_{4} & =\pi D_{1}^{+}\mu^{+}\cos\alpha [\frac{1}{560}({l_{1}}^{7}-{l_{2}}^{7})+\frac{3}{80}({l_{2}}^{2}{l_{1}}^{5}-{l_{1}}^{2}{l_{2}}^{5})+\frac{1}{16}({l_{1}}^{3}{l_{2}}^{4}-{l_{1}}^{4}{l_{2}}^{3})+\frac{1}{80}(l_{1}{l_{2}}^{6}-l_{2}{l_{1}}^{6})]\\ & +\pi D_{1}^{-}\cos\alpha \{\frac{23}{360}{l_{2}}^{6}l_{1}-\frac{11}{36}{l_{1}}^{2}{l_{2}}^{5}+\frac{7}{60}{l_{1}}^{6}l_{2}-\frac{79}{360}{l_{1}}^{5}{l_{2}}^{2}-\frac{17}{72}{l_{1}}^{4}{l_{2}}^{3}+\frac{43}{72}{l_{1}}^{3}{l_{2}}^{4}-\frac{17}{2520}{l_{2}}^{7}\\ & +(\frac{1}{6}{l_{1}}^{5}{l_{2}}^{2}+\frac{1}{2}{l_{1}}^{4}{l_{2}}^{3}+\frac{1}{6}{l_{1}}^{3}{l_{2}}^{4})[\ln(l_{2})-\ln(l_{1})]-\frac{5}{504}{l_{1}}^{7}+\frac{\mu^{-}}{560}({l_{1}}^{7}-{l_{2}}^{7})+\frac{\mu^{-}}{16}({l_{1}}^{3}{l_{2}}^{4}-{l_{1}}^{4}{l_{2}}^{3})\\ & +\frac{\mu^{-}}{80}(l_{1}{l_{2}}^{6}-l_{2}{l_{1}}^{6})+\frac{3\mu^{-}}{80}({l_{2}}^{2}{l_{1}}^{5}-{l_{1}}^{2}{l_{2}}^{5})\} \end{array}$$

$$\begin{array}{cc} h_{5} & =\pi D_{1}^{+}\sin\alpha [\frac{7\mu^{+}}{288}{l_{1}}^{5}{l_{2}}^{5}-\frac{35\mu^{+}}{1728}({l_{1}}^{4}{l_{2}}^{6}-{l_{1}}^{6}{l_{2}}^{4})+\frac{5\mu^{+}}{432}({l_{1}}^{3}{l_{2}}^{7}+{l_{2}}^{3}{l_{1}}^{7})-\frac{5\mu^{+}}{1152}({l_{2}}^{2}{l_{1}}^{5}+{l_{1}}^{2}{l_{2}}^{5})\\ & +\frac{5\mu^{+}}{5184}(l_{1}{l_{2}}^{9}+l_{2}{l_{1}}^{9})+\frac{463}{4320}{l_{2}}^{6}{l_{1}}^{4}+\frac{367}{4320}{l_{1}}^{6}{l_{2}}^{4}+\frac{1}{5184}{l_{1}}^{10}+\frac{131}{181440}{l_{2}}^{10}-\frac{83}{720}{l_{1}}^{5}{l_{2}}^{5}\\ & +\frac{559}{20160}{l_{1}}^{2}{l_{2}}^{8}-\frac{73}{1080}{l_{1}}^{3}{l_{2}}^{7}-\frac{319}{7560}{l_{2}}^{3}{l_{1}}^{7}+\frac{271}{20160}{l_{2}}^{2}{l_{1}}^{8}-\frac{607}{90720}l_{1}{l_{2}}^{9}-\frac{223}{90720}l_{2}{l_{1}}^{9}\\ & -\frac{\mu^{+}}{10368}{l_{1}}^{10}-\frac{1}{10368}\mu^{+}{l_{2}}^{10}]+\pi D_{1}^{-}\mu^{-}\sin\alpha [\frac{5}{432}({l_{1}}^{3}{l_{2}}^{7}+{l_{2}}^{3}{l_{1}}^{7})+\frac{5}{5184}(l_{1}{l_{2}}^{9}+l_{2}{l_{1}}^{9})\\ & -\frac{35}{1728}({l_{1}}^{4}{l_{2}}^{6}-{l_{1}}^{6}{l_{2}}^{4})-\frac{1}{10368}({l_{2}}^{10}+{l_{1}}^{10})+\frac{7}{288}{l_{1}}^{5}{l_{2}}^{5}-\frac{5}{1152}({l_{2}}^{2}{l_{1}}^{8}+{l_{1}}^{2}{l_{2}}^{8})] \end{array}$$

$$\begin{array}{cc} h_{6} & =\pi D_{1}^{+}\sin\alpha \left(\frac{1}{15}{l_{1}}^{5}l_{2}-\frac{7}{15}{l_{2}}^{5}l_{1}-\frac{2}{3}{l_{1}}^{3}{l_{2}}^{3}+\frac{5}{6}{l_{2}}^{4}{l_{1}}^{2}+\frac{1}{6}{l_{1}}^{4}{l_{2}}^{2}-\frac{1}{30}{l_{1}}^{6}+\frac{1}{10}{l_{2}}^{6}\right)\\ & +\pi D_{1}^{-}\sin\alpha \left[\frac{2}{5}\right.{l_{1}}^{5}l_{2}+\frac{1}{60}{l_{2}}^{6}-\frac{1}{30}l{l_{1}}^{6}+\frac{7}{12}{l_{1}}^{4}{l_{2}}^{2}-\frac{4}{3}{l_{1}}^{3}{l_{2}}^{3}-\frac{2}{15}{l_{2}}^{5}l_{1}+\frac{1}{2}{l_{2}}^{4}{l_{1}}^{2}\\ & \left.+{l_{1}}^{4}{l_{2}}^{2}\ln(l_{2})-{l_{1}}^{4}{l_{2}}^{2}\ln(l_{1})\right] \end{array}$$

$$\begin{array}{cc} h_{7} & =\pi \cos\alpha \left(D_{1}^{+}\mu^{+}+D_{1}^{-}\mu^{-}\right)\left[\frac{37}{4320}\left({l_{1}}^{6}{l_{2}}^{5}-{l_{1}}^{5}{l_{2}}^{6}\right)\right.+\frac{37}{36288}\left({l_{1}}^{2}{l_{2}}^{9}-{l_{1}}^{9}{l_{2}}^{2}\right)+\frac{37}{1995840}\left({l_{2}}^{11}-{l_{1}}^{11}\right)\\ & +\frac{37}{181440}\left({l_{1}}^{10}l_{2}-l_{1}{l_{2}}^{10}\right)+\frac{37}{6048}\left({l_{1}}^{4}{l_{2}}^{7}-{l_{1}}^{7}{l_{2}}^{4}\right)\left.+\frac{37}{12096}\left({l_{1}}^{8}{l_{2}}^{3}-{l_{1}}^{3}{l_{2}}^{8}\right)\right] \end{array}$$

$$\begin{array}{cc} h_{8}= & \pi D_{1}^{+}\sin\alpha [\frac{1801}{1935360}({l_{2}}^{8}{l_{1}}^{6}-{l_{2}}^{6}{l_{1}}^{8})+\frac{1801}{830269440}({l_{2}}^{14}-{l_{1}}^{14})+\frac{1801}{1451520}({l_{2}}^{5}{l_{1}}^{9}-{l_{2}}^{9}{l_{1}}^{5})\\ & +\frac{1801}{69189120}(l_{2}{l_{1}}^{13}-{l_{2}}^{13}l_{1})+\frac{1801}{12773376}({l_{1}}^{2}{l_{2}}^{12}-{l_{1}}^{12}{l_{2}}^{2})+\frac{1801}{1935360}({l_{2}}^{10}{l_{1}}^{4}-{l_{2}}^{4}{l_{1}}^{10})\\ & +\frac{1801}{3991680}({l_{2}}^{3}{l_{1}}^{11}-{l_{2}}^{11}{l_{1}}^{3})]\\ & +\pi D_{1}^{-}\mu^{-}\sin\alpha [\frac{431}{12096}({l_{1}}^{7}{l_{2}}^{4}-{l_{1}}^{4}{l_{2}}^{7})+\frac{431}{72576}({l_{1}}^{9}{l_{2}}^{2}-{l_{2}}^{9}{l_{1}}^{2})+\frac{431}{8640}({l_{1}}^{5}{l_{2}}^{6}-{l_{1}}^{6}{l_{2}}^{5})\\ & +\frac{431}{24192}({l_{2}}^{8}{l_{1}}^{3}-{l_{1}}^{8}{l_{2}}^{3})+\frac{431}{362880}({l_{2}}^{10}l_{1}-{l_{1}}^{10}l_{2})+\frac{431}{3991680}({l_{1}}^{11}-{l_{2}}^{11})] \end{array}$$

$$\begin{array}{cc} h_{9} & =\pi D_{1}^{-}\cos\alpha \left\{\frac{59}{360}\left(l_{1}{l_{2}}^{5}-{l_{1}}^{5}l_{2}\right)+\left(\frac{1}{6}{l_{1}}^{4}{l_{2}}^{2}+\frac{1}{6}{l_{1}}^{2}{l_{2}}^{4}+\frac{1}{2}{l_{1}}^{3}{l_{2}}^{3}\right)\left[\ln(l_{1})-\ln(l_{2})\right]\right.+\frac{1}{60}\left({l_{1}}^{6}-{l_{2}}^{6}\right)\\ & \left.+\frac{13}{36}\left({l_{1}}^{4}{l_{2}}^{2}-{l_{1}}^{2}{l_{2}}^{4}\right)\right\} \end{array}$$

$$\begin{array}{cc} h_{10} & =\pi D_{1}^{-}\cos\alpha \cot\alpha \{(\frac{1}{144}{l_{1}}^{6}{l_{2}}^{2}-\frac{1}{24}{l_{1}}^{5}{l_{2}}^{3}+\frac{11}{144}{l_{1}}^{4}{l_{2}}^{4}-\frac{1}{24}{l_{1}}^{3}{l_{2}}^{5}+\frac{1}{144}{l_{1}}^{2}{l_{2}}^{5})[\ln(l_{2})-\ln(l_{1})]\\ & +\frac{101}{120960}({l_{2}}^{8}-{l_{1}}^{8})+\frac{59}{6048}({l_{1}}^{7}l_{2}-l_{1}{l_{2}}^{7})+\frac{11}{288}({l_{1}}^{2}{l_{2}}^{6}-{l_{1}}^{6}{l_{2}}^{2})+\frac{233}{4320}({l_{1}}^{5}{l_{2}}^{3}-{l_{1}}^{3}{l_{2}}^{5})\}\\ & +\pi D_{2}^{+}\sin\alpha \{\frac{1}{30}(1-\mu^{+})({l_{2}}^{5}-{l_{1}}^{5})+(\frac{1}{6}+\frac{\mu^{+}}{9})({l_{1}}^{4}l_{2}-{l_{2}}^{4}l_{1})+(\frac{1}{3}+\frac{7\mu^{+}}{6})({l_{1}}^{2}{l_{2}}^{3}-{l_{1}}^{3}{l_{2}}^{2})\\ & +\mu^{+}[\ln(l_{2})-\ln(l_{1})](\frac{1}{6}l_{1}{l_{2}}^{4}+\frac{1}{6}{l_{1}}^{4}l_{2}-\frac{1}{2}{l_{1}}^{3}{l_{2}}^{2}-\frac{1}{2}{l_{1}}^{2}{l_{2}}^{3})\}\\ & +\pi D_{2}^{-}\sin\alpha \{\frac{1}{30}(1-\mu^{+})({l_{2}}^{5}-{l_{1}}^{5})+(\frac{1}{6}+\frac{\mu^{+}}{9})({l_{1}}^{4}l_{2}-{l_{2}}^{4}l_{1})+(\frac{1}{3}+\frac{7\mu^{+}}{6})({l_{1}}^{2}{l_{2}}^{3}-{l_{1}}^{3}{l_{2}}^{2})\\ & +\mu^{+}[\ln(l_{2})-\ln(l_{1})](\frac{1}{6}l_{1}{l_{2}}^{4}+\frac{1}{6}{l_{1}}^{4}l_{2}-\frac{1}{2}{l_{1}}^{3}{l_{2}}^{2}-\frac{1}{2}{l_{1}}^{2}{l_{2}}^{3})\} \end{array}$$

$$\begin{array}{cc} h_{11} & =\pi \left(D_{2}^{+}+D_{2}^{-}\right)\sin\alpha \left\{\left(\frac{1}{12}{l_{2}}^{3}+\frac{1}{12}{l_{1}}^{3}-\frac{1}{4}{l_{1}}^{2}l_{2}-\frac{1}{4}l_{1}{l_{2}}^{2}\right)\left[\ln(l_{2})-\ln(l_{1})\right]\right.\\ & \left.+\frac{3}{4}{l_{2}}^{2}l_{1}-\frac{5}{36}{l_{2}}^{3}+\frac{5}{36}{l_{1}}^{3}-\frac{3}{4}l_{2}{l_{1}}^{2}\right\} \end{array}$$

$$\begin{array}{cc} p_{1} & =2\pi N_{T}\cos\alpha \left[\frac{1}{12}\left({l_{1}}^{4}l_{2}-{l_{2}}^{4}l_{1}\right)+\frac{1}{6}\left({l_{2}}^{3}{l_{1}}^{2}-{l_{2}}^{2}{l_{1}}^{3}\right)+\frac{1}{60}\left({l_{2}}^{5}-{l_{1}}^{5}\right)\right]\\ & +2\pi M_{T}\sin\alpha \left[\frac{1}{6}\left({l_{1}}^{3}l_{2}-{l_{2}}^{3}l_{1}\right)\right.\left.+\frac{1}{12}\left({l_{2}}^{4}-{l_{1}}^{4}\right)\right] \end{array}$$

$$p_{2}=2\pi N_{T}\sin\alpha \left[\left.\frac{17}{20160}\left({l_{2}}^{8}-{l_{1}}^{8}\right)+\frac{17}{3360}\left({l_{1}}^{7}l_{2}-l_{1}{l_{2}}^{7}\right)+\frac{17}{1440}\left({l_{1}}^{2}{l_{2}}^{6}-{l_{1}}^{6}{l_{2}}^{2}{l_{1}}^{5}{l_{2}}^{3}-{l_{1}}^{3}{l_{2}}^{5}\right)\right]\right.$$

$$q_{2}=2\pi q\sin\alpha \left(\frac{1}{120}{l_{2}}^{6}-\frac{1}{30}l_{1}{l_{2}}^{5}+\frac{1}{24}{l_{2}}^{4}{l_{1}}^{2}-\frac{1}{120}{l_{1}}^{6}+\frac{1}{30}{l_{1}}^{5}l_{2}-\frac{1}{24}{l_{1}}^{4}{l_{2}}^{2}\right)$$

$$\eta_{0}=h_{11}{h_{2}}^{2}-4h_{11}h_{3}h_{6}-p_{1}{h_{2}}^{2}+h_{2}h_{4}p_{2}+4p_{1}h_{3}h_{6}-2p_{2}h_{6}h_{9}$$

$$\eta_{1}=2h_{10}{h_{2}}^{2}-4p_{2}h_{1}h_{6}-8h_{10}h_{3}h_{6}-q_{2}{h_{2}}^{2}-2h_{2}h_{4}h_{9}+2p_{2}h_{2}h_{5}+2h_{3}{h_{4}}^{2}+4q_{2}h_{3}h_{6}+2h_{6}{h_{9}}^{2}$$

$$\eta_{2}=3h_{7}{h_{2}}^{2}-3h_{1}h_{2}h_{4}+6h_{1}h_{6}h_{9}-3h_{2}h_{5}h_{9}+6h_{3}h_{4}h_{5}-12h_{3}h_{6}h_{7}$$

$$\eta_{3}=4{h_{1}}^{2}h_{6}-4h_{1}h_{5}h_{2}+4h_{8}{h_{2}}^{2}+4h_{3}{h_{5}}^{2}-16h_{3}h_{6}h_{8}$$

## Appendix C

**Table A1:** The dimensionless maximum deflection *W*_max_ of conical shell under thermal load.

Thermal Load (°C)	Variation Solution		Finite Element Simulation	
	Fully Fixed	Simply Supported	Fully Fixed	Simply Supported
30	0.0125667	0.0154956	0.0136497	0.0163698
40	0.0255390	0.0310316	0.0274292	0.0328225
50	0.0387499	0.0466039	0.0410009	0.0493505
60	0.0521225	0.0621889	0.0549021	0.0659476
70	0.0656242	0.0778072	0.0689209	0.0826093
80	0.0792377	0.0933818	0.0830539	0.0993271
90	0.0929524	0.1089619	0.0973030	0.1161163
100	0.1067607	0.1245250	0.1116606	0.1329716
110	0.1206568	0.1400318	0.1261353	0.1498695
120	0.1346357	0.1557335	0.1422767	0.1668087

**Table A2:** The dimensionless maximum deflection *W*_max_ of conical shell under mechanical load.

Mechanical Load (MPa)	Variation Solution		Finite Element Simulation	
	Fully Fixed	Simply Supported	Fully Fixed	Simply Supported
0.1	0.0447111	0.0532963	0.0431253	0.0525356
0.2	0.0914913	0.1095889	0.0872441	0.1087107
0.3	0.1405517	0.1692484	0.1326811	0.1673154
0.4	0.1978796	0.2326697	0.1789938	0.2303863
0.5	0.2393931	0.3002344	0.2264006	0.2978788
0.6	0.2809066	0.3722855	0.2749376	0.3709397
0.7	0.3316193	0.4490180	0.3246377	0.4496797
0.8	0.3823320	0.5303973	0.3749333	0.5330559
0.9	0.4342266	0.6160686	0.4280871	0.6176847
1.0	0.4861212	0.7054740	0.4822724	0.7289896
